# Determination of pesticide residues in urine by chromatography-mass spectrometry: methods and applications

**DOI:** 10.3389/fpubh.2024.1336014

**Published:** 2024-06-12

**Authors:** Willian Garcia Birolli, Fernando Mauro Lanças, Álvaro José dos Santos Neto, Henrique C. S. Silveira

**Affiliations:** ^1^Molecular Oncology Research Center, Barretos Cancer Hospital, São Paulo, Brazil; ^2^Chromatography Group, São Carlos Institute of Chemistry, University of São Paulo, São Paulo, Brazil

**Keywords:** biomonitoring, agrochemical, occupational exposure, risk assessment, GC–MS/MS, LC–MS/MS, toxicity, health outcome

## Abstract

**Introduction:**

Pollution has emerged as a significant threat to humanity, necessitating a thorough evaluation of its impacts. As a result, various methods for human biomonitoring have been proposed as vital tools for assessing, managing, and mitigating exposure risks. Among these methods, urine stands out as the most commonly analyzed biological sample and the primary matrix for biomonitoring studies.

**Objectives:**

This review concentrates on exploring the literature concerning residual pesticide determination in urine, utilizing liquid and gas chromatography coupled with mass spectrometry, and its practical applications.

**Method:**

The examination focused on methods developed since 2010. Additionally, applications reported between 2015 and 2022 were thoroughly reviewed, utilizing Web of Science as a primary resource.

**Synthesis:**

Recent advancements in chromatography-mass spectrometry technology have significantly enhanced the development of multi-residue methods. These determinations are now capable of simultaneously detecting numerous pesticide residues from various chemical and use classes. Furthermore, these methods encompass analytes from a variety of environmental contaminants, offering a comprehensive approach to biomonitoring. These methodologies have been employed across diverse perspectives, including toxicological studies, assessing pesticide exposure in the general population, occupational exposure among farmers, pest control workers, horticulturists, and florists, as well as investigating consequences during pregnancy and childhood, neurodevelopmental impacts, and reproductive disorders.

**Future directions:**

Such strategies were essential in examining the health risks associated with exposure to complex mixtures, including pesticides and other relevant compounds, thereby painting a broader and more accurate picture of human exposure. Moreover, the implementation of integrated strategies, involving international research initiatives and biomonitoring programs, is crucial to optimize resource utilization, enhancing efficiency in health risk assessment.

## 1 Introduction

Pollution currently poses a significant threat to humanity, necessitating the development of various biomonitoring techniques (1). Humans are exposed to a multitude of contaminants through air, dust, water, food, and personal care products, entering our bodies via ingestion, inhalation, or dermal absorption (2). Such exposures to complex combinations can lead to serious adverse effects, even when individual substances in mixtures are below safety limits, highlighting the need for a comprehensive understanding of combined human exposure for public health initiatives (3).

Several countries have established regulatory institutions to control and prevent pollution (4). However, only a few, including Germany, France, Israel, United States, and Canada, have successfully integrated human biomonitoring studies with health data (5). In this regard, health risk assessments play a crucial role in informing decision-makers on protecting human health and the environment (6, 7), with pesticide residues in food emerging as a particularly relevant concern (8).

While pesticides offer undeniable benefits to humanity, such as increased production and improved quality of life (9), these formulations also represent some of the oldest and most widely used environmental contaminants, posing severe toxicity issues necessitating decontamination strategies (10, 11). Risks associated with pesticide exposure range from short-term effects, like skin irritation and headaches, to chronic impacts such as cancer, Parkinson’s and Alzheimer’s diseases, asthma, and diabetes (12). Although the toxicological mechanisms are not fully elucidated, chronic pesticide exposure may involve different interactions than those targeting the pesticides’ primary intended use (13).

There are no population groups in the world unexposed to these compounds (14), but understanding these risks is challenging due to several factors, including the duration and level of exposure, type of contact, toxicity, persistence, and environmental characteristics (15). Therefore, studies employing direct and indirect determination methods for pesticide exposure have been conducted (16), with human biomonitoring enabling the measurement of personal exposure to specific chemicals, including both unmetabolized and metabolized compounds (17).

This is typically achieved through the analysis of biological samples like blood, plasma, serum, and urine, frequently complemented with oral fluid, hair, and nails, employing methods that must be fast, sensitive, and specific, emphasizing miniaturization and automation (18, 19). Technological advancements have led to increasingly sensitive detection methods capable of measuring pesticides accurately in parts per billion (ng·mL^−1^, or μg·kg^−1^), even with less expensive instrumentation and simplified approaches (20). Mass spectrometry, particularly in combination with chromatography, remains among the most selective, sensitive, and rapid analytical techniques for investigating organic contaminants in different sample matrices (21).

Urine stands out as the primary matrix in biomonitoring programs and cohort studies due to its non-invasive, painless, and easy collection process, making it ideal for studies requiring large participant numbers (22–24). However, these samples present challenges due to various interferents, prompting the development of numerous methods to address these issues (25). Taking pyrethroids as an illustration, reports of contamination in sediments, water, and crops have outnumbered those concerning humans (26).

Both endogenous and exogenous substances in biological samples contribute to matrix effects (MEs), a complex phenomenon with compound- and system-specific characteristics, requiring diverse management strategies (25). Depending on the sample matrix and analyte concentration, different sample preparation techniques may be necessary, such as dilution, protein precipitation, and extraction (27).

**Figure 1:**
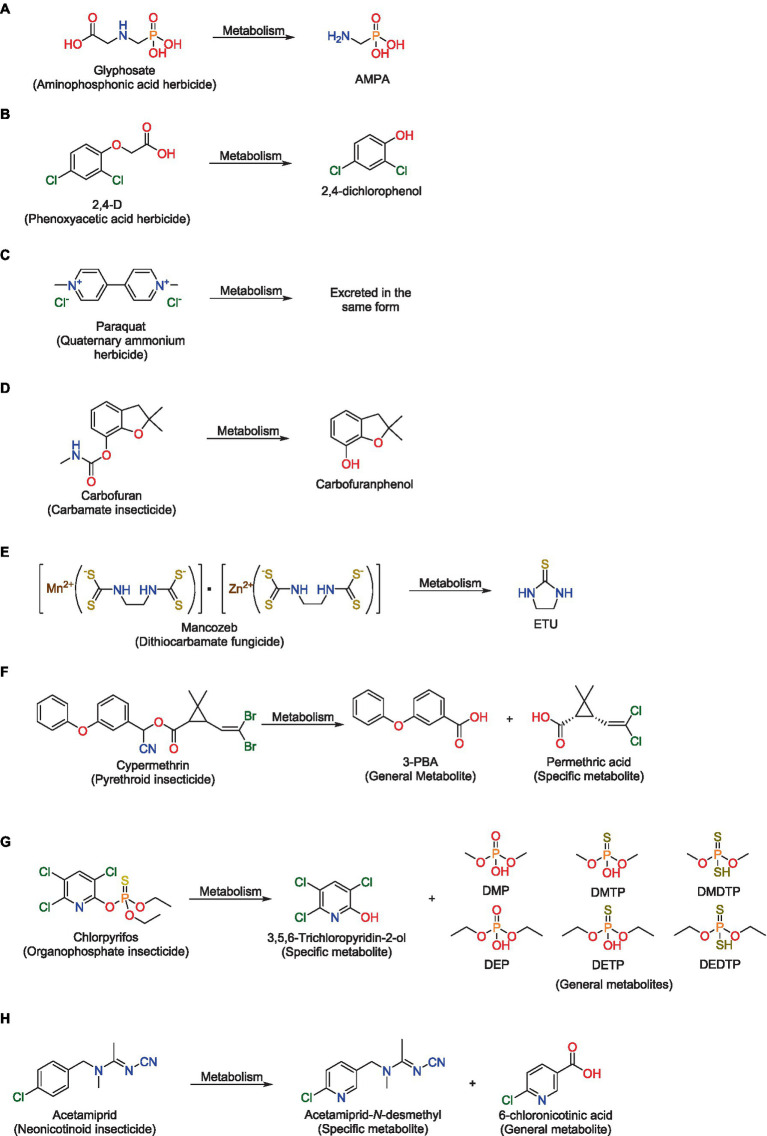
Few examples of active ingredients and their exposure biomarkers. **(A)** Glyphosate, **(B)** 2,4-D, **(C)** Paraquat, **(D)** Carbofuran, **(E)** Mancozeb, **(F)** Cypermethrin, **(G)** Chlorpyrifos, **(H)** Acetamiprid.

Understanding the environmental fate of pesticides, metabolites, and excreted forms is crucial for contamination assessment (28). In this regard, there are different groups of exposure biomarkers with general or specific associations with known contaminants (Figure 1). Different chemical classes, such glyphosate and 2,4-D frequently detected in urine, can be highlighted along with their respective metabolites AMPA and 2,4-dichlorophenol (Figures 1A,B). In contrast, paraquat is mainly excreted without modification (Figure 1C), while many active ingredients such as carbofuran, mancozeb, cypermethrin, chlorpyrifos, and acetamiprid (Figures 1D–H) are primarily monitored in humans through their biotransformation metabolites.

Some active ingredients, such as highly polar or ionic compounds like glyphosate, glufosinate, quaternary ammonium, and phenoxy acids, are often excluded from multi-residue methods and are instead addressed in more specialized methods (29). In this regard, it is crucial to highlight the widespread dispersion of these active ingredients in the environment, facilitated by their increased water solubility, emphasizing the need for new and broader analytical strategies (29).

New methodologies have emerged prioritizing microextraction techniques, which demand less solvent, labor, cost, and environmental impact (30). Consequently, traditional techniques like Liquid–Liquid Extraction (LLE) and Solid Phase Extraction (SPE), known for their time-consuming multistage operations and high solvent consumption, are no longer considered optimal (31). Recognizably, the analytical chemistry field now emphasizes eco-friendly alternatives and reduced use of harmful chemicals (32).

More recently, SPE cartridges utilizing few milligrams of stationary phase, have evolved into a well-established technique with new sorbents, experimental setups, and automation, facilitating widespread adoption and enabling various applications with reduced solvent use (33). Additionally, Solid-Phase Microextraction (SPME), involving thin fibers coated with a sorption material, and its derivatives like packed-in-tube solid-phase microextraction (IT-SPME), have gained attention (34, 35).

Another widely explored technique was the QuEChERS extraction method, known for its acronym representing Quick, Easy, Cheap, Effective, Rugged, and Safe, extensively utilized due to its efficient removal of matrix effects and high recovery rates of target analytes (36). Similarly, Dispersive Liquid–Liquid Microextraction (DLLME), a miniaturized LLE technique, found application in biological fluids (37), as Solidification of Floating Organic Drop Microextraction (SFODME), which involves solidifying a floating organic droplet (38). Nevertheless, exploring robotic and on-flow extraction methods was explored to mitigate labor-intensive and error-prone procedures (39).

Method validation is undeniably crucial in analytical chemistry. Therefore, several organizations have published guidance documents covering limits of detection and quantification, selectivity, specificity, confirmation of identity, robustness, linearity, precision, accuracy, trueness, matrix effect, quality control, and uncertainty, all vital for quality assurance (40, 41).

Thus, this review focuses on discussing literature concerning residual pesticide determination in urine, employing liquid and gas chromatography coupled with mass spectrometry from 2010 to 2022, emphasizing recent developments and trends. Furthermore, it delves into applications from 2015 to 2022, illustrating the identification of impacts and correlations of pesticide exposure with human health facilitated by these methodologies.

## 2 Scope of research

The literature search encompassed articles indexed in the Web of Science database from 2010 to 2022. Initial queries focused on key terms: “pesticide+urine+determination,” yielding 362 research articles; “pesticide+urine quantification,” yielding 251 research articles; and “pesticide+urine+chromatography,” yielding 621 research articles. Subsequently, these results underwent screening based on title and abstract criteria, targeting articles employing chromatography-mass spectrometry systems for determinations. The refined literature was then categorized by the authors into Methods and Techniques, delineating method development in the utilization of Gas Chromatography–Mass Spectrometry (GC–MS, 19 articles), Gas Chromatography–Mass Spectrometry Tandem (GC–MS/MS, 11 articles), and Liquid Chromatography-Mass Spectrometry Tandem (LC–MS/MS, 46 articles). Moreover, a separated category titled Applications was established aiming to derive meaningful and conclusive results for assessing exposure and health effects, comprising literature published between 2015 and 2022, and focusing on General Population Exposure (18 articles), Occupational Exposure (21 articles), and Reproductive Disorders and Early Stages of Life (38 articles).

## 3 Methods and techniques

**Figure 2:**
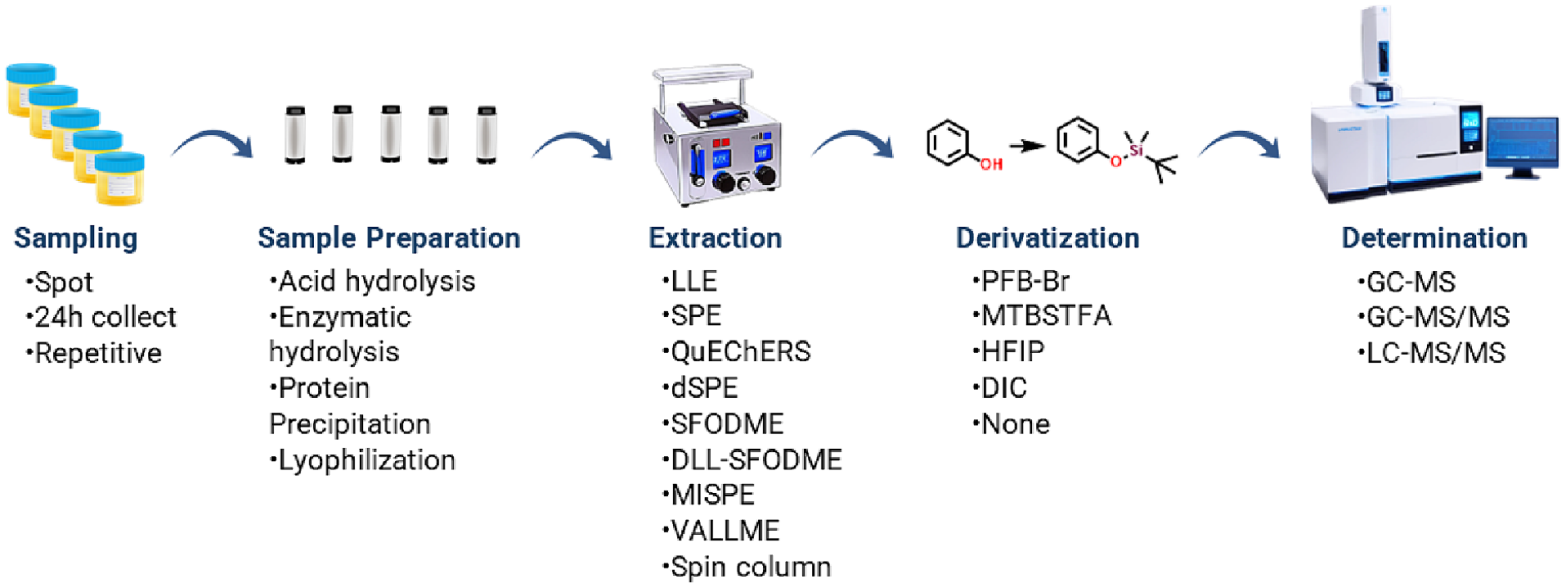
General steps of methods for human biomonitoring by urine analysis.

The methods outlined were categorized based on the instrumentation employed for analysis: Gas Chromatography–Mass Spectrometry (GC–MS), Gas Chromatography–Mass Spectrometry Tandem (GC–MS/MS), and Liquid Chromatography-Mass Spectrometry Tandem (LC–MS/MS). Refer to Figure 2 for an overview of the typical workflow incorporating these prevalent techniques.

### 3.1 GC–MS

GC–MS has been employed for quantification of pesticide residues in urine, as outlined in Table 1. While this technique exhibits reduced selectivity compared to GC–MS/MS, it stands as a cost-effective alternative for determining residues from various active ingredients in multi-residue methods, including metabolites of organophosphates, pyrethroids, triazoles, and aminophosphonic acids (48, 55, 60).

**Table 1:** Methods for pesticide residue determination in urine employing GC–MS.

Analyte (LOQ in ng·mL^−1^)	Urine volume and sample preparation	Extraction	Chromatographic column	Accuracy (%)^a^	Precision (%)^a^	References
4 DAPs (0.2–0.5).	2.5 mL. Acid hydrolysis with HCl.	Sodium disulfite and NaCl addition. LLE with diethyl ether and acetonitrile. Derivatization with PFB-Br. Clean-up with primary-secondary amine and florisil.	RTX-65 column (30 m × 0.25 mm, 0.25 μm).	63–102	7–37	(42)
6 DAPs (0.25–2.5).	2.5 mL. Acid hydrolysis with HCl.	LLE with acetonitrile and diethyl ether. Derivatization with PFB-Br. Additional LLE with water and hexane addition.	HP-5MS capillary column (60 m × 0.25 mm, 0.25 μm).	92–103	1.0–15	(43)
2 DAPs (10).	1.0 mL.	MISPE using 4-vinylpiridine and ethylene glycol dimethacrylate. Derivatization with PFB-Br.	RTX-5MS column (30 m × 0.25 mm, 0.25 μm).	83–111	7–19	(44)
1 organophosphate pesticide and 1 specific metabolite: (0.0129–0.0156). Ch	5.0 mL.	SFODME with 2-dodecanol.	DB-5MS (30 m × 0.32 mm, 0.25 μm).	100–110	1–14	(45)
2 specific organophosphate metabolites: (1.0–2.0)	500 μL. Enzymatic hydrolysis.	LLE with ethyl acetate. Derivatization with MTBSTFA.	RTX-65 column (30 m × 0.25 mm, 0.25 μm).	68–118	2–11	(46)
15 metabolites of organophosphorus compounds, including insecticides, flame retardants, plasticizers, and moth repellents (0.8–3.8)	5.0 mL. Acid hydrolysis with HCl.	SPE Isolute ENV+. Derivatization with MTBSTFA.	DB-5MS (30 m × 0.25 mm, 0.25 μm)	0–17	1–19	(47)
3 organophosphates (100). 2 phosphonic acid herbicides (100–5000)	0.2 mL.	Extraction with mixed-mode C–C18 monolithic spin column. Derivatization with MTBSTFA.	HP-5MS capillary column (30 m × 0.25 mm, 0.25 μm).	97–110	6–13	(48)
3 organophosphates 2 phosphonic acid herbicides (100–500)	0.2 mL.	Extraction with mix-mode TiO-C18 monolithic spin column. Derivatization with MTBSTFA.	HP-5MS capillary column (30 m × 0.25 mm, 0.25 μm).	97–108	6–12	(49)
2 bipyridyl quaternarium ammonium herbicides. 1 organophosphate	0.2 mL.	Monolitic spin column extraction with Monospin C18.	HP-5MS capillary column (30 m × 0.25 mm, 0.25 μm)	51–106	3–15	(50)
4 pyrethroids: (1.5–60.6)	2.0 mL.	Salting-out assisted liquid–liquid extraction.	ZB-5 MS, (30 m × 0.25 mm, 0.25 μm)	75–99	2–9	(51)
4 pyrethroids: (5–20)	2.0 mL.	Salting-out assisted liquid–liquid extraction with NaCl and phosphate buffer solution. DLLME-SFO, in this case, 1-undecanol.	ZB-5MS column (30 m × 0.25 mm, 0.25 μm)	68–103	0–3	(52)
2 pyrethroids metabolites:	2.0 g of urine absorber from diaper. Acid hydrolysis with HCl.	Extraction from diaper with acetone. SPE Extraction with tert-butyl methyl ether. Derivatization with HFIP.	RTX-65 column (30 m × 0.25 mm., 0.25 μm)	50–118	4–14	(53)
7 pyrethroid metabolites (0.2).	2.5 mL. Enzymatic hydrolysis.	SPE with SampliQ C18 cartridges. Derivatization using HFIP and DIC.	DB-5MS (30 m × 0.25 mm, 0.25 μm).	81–104	4–11	(54)
7 triazole fungicides (10–30).	1.0 mL. Enzymatic hydrolysis.	VALLME with toluene.	Rtx-5MS (30 m × 0.25 mm × 0.25 μm).	101–119	7–19	(55)
15 organochlorine pesticides.	10.0 mL.	Membrane-protected stir-bar supported micro- SPE employing double layered hydroxide/graphene hybrid as sorbent	RXI-5 Sil MS column (30 m × 0.25 mm × 0.25 μm).	84–100	3–10	(56)
2 Organophosphate. 1 Organophosphate metabolite. 2 Triazines. 4 Pyrethroids. 1 Bromopropylate. (7–57)	5.0 mL.	DLLME-SFO employing the deep eutectic prepared with menthol and phenylacetic acid.	HP-1 column (30 m × 0.25 mm × 0.25 μm).	79–96	3–8	(57)
5 Organophosphate 1 Carbamate. 2 Pyrethroid. 1 Insect growth regulator. (10)	2.0 mL.	QuEChERS with magnesium sulfate and sodium acetate. d-SPE with lipid EMR sorbent.	DB-35 ms column (15 m × 0.25 mm, 0.25 μm).	90–105	2–19	(58)
5 Carbamates. 1 Carbamate metabolite. 4 Organophsophates. 1 Organochlorine. (2.5–5.0)	0.1 mL. Dilution.	DPX-RP with styrene-divinylbenzene.	RTX-5MS (30 m × 0.25 mm × 0.25 μm).	63–118	5–13	(59)
Metabolites: 1 Carbamate. 6 Organophosphates. 7 Pyrethroids. (0.1–0.3)	2.0 mL. Acid hydrolysis with HCl.	LLE with hexane. Derivatization with MTBSTFA.	RTX-35 column (30 m × 0.25 mm × 0.25 μm).	89–103	1–5	(60)

a It is important to note that different approaches have been employed for determination accuracy and precision. This table was elaborated for description purposes, please check the respective cited articles for details.

Dialkylphosphates (DAPs), general metabolites of organophosphate insecticides, have been the subject of investigation in several studies utilizing GC–MS (43). Given their considerable solubility in water, extraction techniques utilizing solvents and solid phases with significant polarity have been extensively employed, followed by derivatization for analysis, as evidenced in different studies (43, 44).

A method specifically devised for the determination of four DAPs [dimethylphosphate (DMP), dimethylthiophosphate (DMTP), diethylphosphate (DEP), and diethylthiophosphate (DETP)] was developed for biomonitoring studies, employing LLE, derivatization with 2,3,4,5,6-pentafluorobenzyl bromide (PFB-Br), and clean-up with primary-secondary amine and florisil. In this study, 25 unexposed and 25 occupationally exposed volunteers were assessed, yielding a detection frequency ranging from 88 to 100% (42). Subsequent investigations expanded the scope to include the determination of six DAPs (43).

Various alternative extraction methods have been explored in analytical research. For instance, Molecularly Imprinted Solid Phase Extraction (MISPE) using 4-vinyl pyridine as the functional monomer and ethylene glycol dimethacrylate as cross-linker was developed for determining DETP and diethyldithiophosphate (DEDTP), indicating potential for further investigation (44).

Another assessed extraction method was Solidification of Floating Organic Drop Microextraction (SFODME) with 2-dodecanol for analyzing the organophosphate chlorpyrifos (45). In another method, LLE with subsequent N-(tert-butyldimethylsilyl)-N-methyltrifluoroacetamide (MTBSTFA) derivatization was developed for quantifying specific metabolites of organophosphate pesticides, 3-methyl-4-nitrophenol and 4-nitrophenol, revealing higher contamination levels in workers compared to the general population (46).

Addressing a diversity of environmental contaminants from the organophosphate class, a method was developed with SPE using Isolute ENV+ and derivatization with MTBSTFA for determining 15 organophosphorus metabolites found in insecticides, flame retardants, plasticizers, and moth repellents, with notable compounds being DMP, DEP, and 2,5-dichlorophenol (47).

In the use class of herbicides, the aminophosphonic acid glyphosate is a widely employed active ingredient being the focus of extensive research, including with monolithic spin-columns. This pesticide was targeted using a C–C18 column extraction method, along with its co-formulant glufosinate, and the organophosphates fenitrothion, malathion, and phenthoate (48), later, a TiO-C18 column was also explored for this purpose (49). In another approach, a method employing reduction with sodium borohydride and C18 monolithic spin column extraction was developed for determining the quaternary ammonium herbicides diquat and paraquat, alongside fenitrothion (50).

In the case of pyrethroids, such as bifenthrin, permethrin, β-cyfluthrin, and fenvalerate, these compounds were quantified using a salting-out assisted LLE method. This approach offered simplicity and environmental friendliness, eliminating the need for specialized equipment during sample preparation (51). Additionally, a combination of salting-out assisted LLE followed by Dispersive Liquid–Liquid Microextraction Based on Solidification of Floating Organic Drop (DLLME-SFO) with 1-undecanol was proposed for different matrices (52).

While previous methods focused on the parent active ingredients, there has been a shift towards determining the general metabolite 3-phenoxybenzoic acid (3-PBA) due to its high excretion rate. Consequently, two pyrethroid metabolites, trans-chrysanthemum dicarboxylic acid and 3-PBA, were identified in diaper samples using acetone, followed by a subsequent extraction with tert-butyl methyl ether and derivatization with 1,1,1,3,3,3-Hexafluoroisopropanol (HFIP) (53).

A more comprehensive approach to pyrethroid assessment involved analyzing 7 metabolites using SPE with C18 cartridges, followed by derivatization with HFIP and N,N′-Diisopropylcarbodiimide (DIC). Furthermore, permethric acid, 3-PBA, and 4-fluoro-3-phenoxybenzoic acid were detected in 100% of the samples from 10 children (54).

For triazole fungicides, an interesting Vortex-Assisted Liquid–Liquid Microextraction (VALLME) method was employed, and active ingredients were detected in all 21 exposed volunteers (55). Moreover, another innovative extraction method using membrane-protected stir-bar-supported micro-solid-phase extraction, characterized with double-layered hydroxide/graphene, was explored for 15 organochlorine pesticides, demonstrating its potential as a pioneering strategy (56).

Several multiresidue methods have been developed to assess exposure to mixtures of active ingredients across various chemical classes. One such approach utilized DLLME-SFO extraction to quantify nine active ingredients from diverse categories (57). In another strategy, the determination of five organophosphates, one carbamate, two pyrethroids, and one insect growth regulator was proposed using QuEChERS extraction followed by Dispersive Solid Phase Extraction (d-SPE) with Enhanced Matrix Removal-Lipid sorbent (58).

In addition, the use of Disposable Pipette Extraction with reverse phase styrene divinylbenzene (DPX-RP) was suggested, with a future aim at automation for high-throughput analyses. This method successfully determined 5 carbamates, 1 carbamate metabolite, and 4 organophosphates (59). This focus on multi-residue analyses was evident in different studies, for example, 13 metabolites from various chemical classes were assessed using LLE with hexane and derivatization with MTBSTFA. As a proof of concept, detection frequencies ranging from 3 to 81% were observed across 30 actual samples (60).

In summary, GC–MS methods were developed to assess pesticide exposure across different chemical and use classes, with emphasis on commonly employed active ingredients (57). Initially, methods were tailored for specific classes such as organophosphates, pyrethroids, organochlorines, or triazoles, before multiresidue strategies were adopted to provide a broader overview (42, 51, 56).

Given that some methods did not employ enzymatic deconjugation, either by enzymatic or acid hydrolysis, this procedure is typically recommended for pesticide metabolites, although most active ingredients excreted in unchanged form may not require this step (49, 53, 54, 60).

Pursuing lower limits of quantification (LOQs) with enhanced accuracy and precision, sample preparation methods initially targeted specific properties of analytes chemical classes before broader methods were explored. For instance, SPE with Isolute ENV+ was effective for hydrophilic compounds, while QuEChERS, DPX-RP, or LLE were suitable for hydrophobic analytes (47, 58–60). Furthermore, various derivatization agents were employed based on the nature of the exposure biomarkers, including PFB-Br, MTBSTFA, and HFIP with DIC (44, 46, 54).

In an advanced strategy, GC–MS/MS has captured growing interest following recent progress that have expanded its availability while reducing acquisition costs. This technology offered improved selectivity and lower limits of quantification compared to GC–MS, becoming widely employed (61).

### 3.2 GC–MS/MS

Approaches employing GC–MS/MS have been developed to target a wide number of analytes in multi-residue methods (Table 2). For example, a method was established for the determination of 8 pyrethroid metabolites using LLE with hexane, followed by derivatization with MTBSTFA. Subsequently, this method was applied to biomonitor 38 individuals from the general population, achieving quantification frequencies ranging from 5 to 100% (62).

**Table 2:** Methods for pesticide residue determination in urine employing GC–MS/MS.

Analyte (LOQ in ng·mL^−1^)	Urine volume and sample preparation	Extraction	Chromatographic conditions	Recovery (%)^a^	Precision (%)^a^	References
8 Pyrethroid metabolites (0.01)	5.0 mL. Acid hydrolysis with HCl.	LLE with hexane. Derivatization with MTBSTFA.	HP-5MS column (60 m × 0.25 mm, 0.25 μm).	91–104	7–11	(62)
9 Pyrethroids metabolites (0.05–0.31)	2.0 mL. Acid hydrolysis with HCl.	LLE with MTBE. Method 1: Derivatization with BSTFA and TMS. Method 2: Derivatization with HFIP and DIC	Method 1: DB-5 ms column. Method 2: Rtx-65 column.	32–116	2–18	(63)
6-Chloronicotinic acid, neonicotinoids metabolites (0.23). 3-PBA, pyrethroids metabolites (0.15).	2.0 mL. Acid hydrolysis with HCl.	LLE with MTBE. Derivatization with BSTFA.	RXI–5 ms (30 m × 0.25 mm × 0.25 μm).	97–104	4–6	(64)
5 organophosphate metabolite and 3 pyrethroids metabolites (0.25–2.5)	10.0 mL. Acid hydrolysis with HCl.	LLE with hexane and isopropanol. The aqueous phase was employed to SPE with WCX cartridges. Derivatization of the SPE elute with MTBSTFA. The organic layer of LLE was put together with the SPE elute after derivatization.	HP-5MS column (30 m × 0.25 mm, 0.25 μm).	54–83	1–9	(65)
7 Endocrine disruptors, including 3 pesticide metabolites (0.3–2.0).	1.0 mL. Enzymatic hydrolysis.	SPE Isolute-101. Derivatization with MTBSTFA.	Zebron ZB-5MS (30 m × 0.25 mm, 0.25 μm)	85–109	0–15	(66)
19 Chlorophenol derivatives (0.04–0.1)	2.0 mL. Enzymatic hydrolysis.	SPE with Isolute-101. Derivatization with BSTFA and 1% TMCS.	ZB-XLB (60 m × 0.25 mm, 0.25 μm).	81–119	1–15	(67)
19 Phenolic metabolites of organophosphates, carbamates and other pesticides (0.2–1.1)	1.0 mL. Enzymatic hydrolysis with β -glucuronidase.	SPE with Isolute-101. Derivatization with MTFA.	HP-5 ms (30 m × 0.25 mm, 0.25 μm).	87–117	1–13	(68)
20 phenolic compounds, including pesticides metabolites and other contaminants (0.0007–0.0098)	0.5 mL. Acid hydrolysis with HCl.	LLE with hexane and MTBE. SPE with K_2_CO_3_-treated-silica-gel. Derivatization with BSTFA.	HP-5 ms (15 m × 0.25 mm, 0.25 μm).	73–107	1–19	(69)
Method 1: 2 Carbamates Method 2: 3 Phthalimides 2 Triazoles 1 Carboxamides biomarkers	2.0 mL. Method 1: Nitric acid addition. Method 2: Enzymatic hydrolysis. Nitric acid addition.	Method 1: SLE with Chem Elut cartridge. Derivatization with MSTFA. Method 2: SPE with Strata-X cartridge. Derivatization with MSTFA.	Rtx-PCB (30 m × 0.25 mm, 0.25 μm).	77–102	11–33	(70)
24 Organochlorines 1 Organophosphate and 8 metabolites. 5 Pyrethroids and 6 metabolites. 2 Carbamate metabolites. 4 PCBs. 6 Other pesticides. (0.004–4)	0.5 mL. Acid hydrolysis with HCl.	Method 1, applied for parent pesticides: SPME with polydimethylsiloxane–divinylbenzene. Method 2, applied for metabolites: LLE with acetonitrile–cyclohexane–ethyl acetate (1:1:1, v/v/v). Derivatization with PFB-Br. Additional LLE with hexane.	HP-5 ms (30 m × 0.25 mm, 0.25 μm).	5–154	1–55	(71)
118 Pesticides from different classes (0.003–1.452)	5.0 mL.	SPE with C18 Sep-Pak cartridges (500 mg).	VF-5 ms (30 m × 0.25 mm, 0.25 μm).	61–119	8–26	(72)

a It is important to note that different approaches have been employed for determination accuracy and precision. This table was elaborated for description purposes, please check the respective cited article for details.

Another strategy involved using two distinct analysis and derivatization methods, exemplified in the determination of 9 pyrethroid metabolites. LLE with tert-butyl methyl ether (MTBE) was employed, followed by derivatization with N,O-bis(trimethylsilyl)trifluoroacetamide (BSTFA) and trimethylchlorosilane (TMCS) for method 1, and HFIP/DIC for method 2. For 50 children, detection frequencies between 38 and 100% were observed (63).

Furthermore, the quantification of the neonicotinoid biomarker 6-chloronicotinic acid and the general metabolite of pyrethroids 3-PBA was performed in another method, also utilizing LLE with methyl tert-butyl ether (MTBE), followed by derivatization with BSTFA. Among 30 children, quantification frequencies were 23% for 6-chloronicotinic acid and 93% for 3-PBA (64).

Expanding the scope of multiresidue determinations, a method for determining 5 organophosphates and 3 pyrethroid metabolites employed LLE with hexane and isopropanol, followed by SPE with weak cation exchange cartridges and derivatization with MTBSTFA. Detection frequencies between 25 and 80% were determined through biomonitoring 20 adults and 20 children, showing that different studies presented high quantification frequency for these active ingredients (65).

Broader methodologies were employed to assess environmental contaminants with potential impacts on human health. For instance, the determination of 7 phenolic endocrine disruptors, including 3 pesticide metabolites, utilized SPE with Isolute-101 and derivatization with MTBSTFA (66). Similarly, a method for biomonitoring 19 chlorophenols, including pesticide metabolites, also employed Isolute-101 but derivatization with BSTFA and TMCS (67). Afterwards, a method for determining 19 phenolic compounds, such as specific metabolites of organophosphates, carbamates, and other pesticides, was developed utilizing LLE with MTBE and derivatization with BSTFA, with an emphasis on discerning exposure between active ingredients from the same chemical class (68).

In a comparable approach, a method for analyzing 20 phenolic compounds, including 7 pesticide biomarkers, used LLE extraction with hexane and MTBE, followed by SPE with K_2_CO_3_-treated silica gel and derivatization with BSTFA. In addition, large volume injection was employed for achieving low detection limits. The analysis of 29 participants revealed an 85% detection rate for most analytes, with mean concentrations ranging from 0.01 to 185 ng·mL^−1^ (69).

Urine and hair analysis, employing a similar analytical method, aimed to determine 2 carbamates, 3 phthalimides, 2 triazoles, and 1 carboxamide biomarker using two analysis methods: Supported Liquid Extraction (SLE) with Chem Elut cartridge and SPE with Strata-X cartridge, both followed by derivatization with N-methyl-N-(trimethylsilyl)trifluoroacetamide (MSTFA) (70). GC–MS/MS with negative chemical ionization was utilized for the determination of 24 organochlorines, 1 organophosphate, 8 organophosphate metabolites, 5 pyrethroids, 6 pyrethroid metabolites, 2 carbamate metabolites, 4 PCBs, and 6 other pesticides for urine and hair analysis. While urine remains the primary matrix for biomonitoring, hair analysis represents exciting alternative for biomonitoring (71).

An integrated approach was adopted to develop a comprehensive biomonitoring strategy, utilizing both GC–MS/MS and LC–MS/MS techniques for analyzing 205 active ingredients. This approach included a shared sample preparation method based on Solid Phase Extraction (SPE) employing C18 Sep-Pak cartridges. The GC–MS/MS method encompassed 118 pesticides from diverse classes, while the LC–MS/MS analysis determined another 87 analytes (72).

Despite fewer methods having been described for GC–MS/MS compared to GC–MS and LC–MS/MS during the reviewed period, it is noteworthy that this determination strategy offers increased selectivity and lower limits of quantification compared to GC–MS (73). Therefore, these strengths were leveraged for the development of multi-residue methods encompassing pesticides of different chemical classes and environmental contaminants originated from several consumer products, resulting in methods covering more than one hundred analytes (66, 70–72).

GC–MS/MS offers attractive cost-efficiency in contrast to LC–MS/MS, primarily because the latter’s equipment tends to be more expensive, often necessitating more recent and complex instrumentation than gas chromatography (74–76). However, despite this advantage, GC–MS/MS does have limitations that constrain its application range. This include the need for analyte derivatization, considerations regarding thermal stability, and issues with volatilization, all of which are not required for the use of LC as the inlet method (77).

### 3.3 LC–MS/MS

A diverse range of LC–MS/MS methods have been developed for analyzing various classes of active ingredients. While exposure biomarkers characterized by significant hydrophobic properties have been thoroughly investigated using multi-residue methods covering a wide range of analytes (77, 78), compounds exhibiting notable aqueous solubility required more restricted approaches, particularly regarding the number of analytes included (79, 80). Detailed information regarding these methodologies is provided in Table 3.

**Table 3:** Summary of the approached methods for pesticide determination in urine employing LC–MS/MS.

Method	Analyte (LOQ in ng·mL^−1^)	Urine volume and sample preparation	Extraction	Chromatographic conditions	Recovery (%)^a^	Inter-day precision (%)^a^	Matrix effect (%)^a^	References
UPLC-ESI-TQ	Glyphosate (50). Glufosinate (50). AMPA (50).	0.2 mL. Protein precipitation. Derivatization with acetate/acetic anhydride and trimethyl orthoacetate.	–	Scherzo SS-C18 column (2.0 mm, 150 mm, 3 μm) (A) 10 mM NH_4_HCO_2_ in water and (B) 10 mM NH_4_HCO_2_ in acetonitrile	84–104	1–18	65–90	(81)
UPLC-ESI-TQ	Glyphosate (1000). AMPA (500).	1.0 mL. Lyophilization. Derivatization with FMOC-Cl.	–	BEH C18 column (2.1 × 100 mm, 1.7 μm) (A) Water with 10 mM ammonium acetate and (B) acetonitrile and water (95:5 v/v) with 10 mM ammonium acetate.	104–119	5–13	19–23	(82)
UPLC-Orbitrap	Glyphosate (6). AMPA (2.5).	0.2 mL. Protein precipitation.	Cold-induced phase separation. Hydrophilic pipette tip solid-phase extraction.	Obelisc N column (2.1 mm × 100 mm, 5 μm) (A) Water with 0.1% formic acid and (B) acetonitrile with 0.1% formic acid	78–110	3–9	4–7	(83)
HPLC-ESI-TQ	Glyphosate. AMPA. *N*-acetyl AMPA. *N*-acetyl glyphosate.	0.2 mL. Dilution with aqueous solution of 1% formic acid.	Cleaned up using Oasis MCX 96-well plates.	Hypercarb column (1 mm × 100 mm, 5 μm). (A) Water with 0.5 M Formic acid and 5 mM medronic acid 50%. (B) 0.5 M Formic acid and 5 M Medronic acid in H2O	93–120	4–10	Not significant	(84)
UPLC-ESI-TQ	GLYP (100) GLUF (100) Diquat (100) Paraquat (200)	0.1 mL.	Protein precipitation with acetonitrile followed by backwashing with dichloromethane.	-Agilent ZORBAX SB-Aq column (2.1 × 100 mm, 1.8 μm particle). (A) Water with 15 mM heptafluorobutyric acid and (B) acetonitrile.	96–113	1–10	6–73	(85)
HPLC-ESI-TQ	Chlormequat (0.1)	0.1 mL.	SPE with Isolute HCX-Q.	Atlantis HILIC column (2.1 mm × 150 mm, 3 μm). (A) Water with 0.05 M acetic acid/ammonium acetate buffer, and (B) acetonitrile with 0.05 M acetic acid/ammonium acetate buffer.	70–84	5–6	Not determined	(86)
HPLC-APCI-SQ	ETU (0.3)	2.0 mL.	Diatomaceous earth extraction columns ChemElut 1,003.	Genesis C18 (4.6 × 250 mm, 4 μm). Isocratic separation with water and methanol (80:20) with 0.1% formic acid.	93	14	Not determined	(87)
UPLC-APCI-TQ	ETU (0.5)	0.5 mL. Alkaline hydrolysis with NaOH.	Online on-column extraction.	Bidimensional separation with two Genesis® Lightn AQ columns (C18, 4.6 × 100 mm, 4 μm) (A) Water with 0.1% formic acid and (B) methanol with 0.1% formic acid	87–120	19	Not determined	(88)
UPLC-ESI-TQ	5-Hydroxythiabendazole, a thiabendazole metabolite (0.13).	0.5 mL. Hydrolysis with β-glucuronidase.	SPE with ISOLUTE®-96 ENV+ plate.	Poroshell 120EC-C18 column (4.6 × 250 mm, 2.7 μm) (A) Water with 0.1% formic acid and (B) methanol with 0.1% formic acid.	94–120	9	1	(89)
HPLC-APCI-TQ	Captan metabolite Tetrahydrophthalimide (1.9). Folpet metabolite phthalimide (3.75).	3.0 mL.	SPE with OASIS cartridge.	C18 Zorbax Eclipse Plus column (4.6 × 150 mm, 3.5 μm). (A) Water and (B) acetonitrile.	87–111	8–28	Not determined	(90)
HPLC-APCI-TQ	7 Atrazine metabolites (0.05–0.2).	1.0 mL.	Cation-exchange SPE with Strata X-C cartridge.	Gemini C6-Phenyl (100 mm × 4.6 mm, 3 μm). (A) Water with 0.1% formic acid, and (B) methanol with 0.1% formic acid	98–101	2–9	Not determined	(91)
HPLC-TQ	4 DAPs: DEP, DETP, DMP, and DMTP (0.3–1.2).	1.0 mL.	Two sequential SPE procedure using Oasis WAX cartridge.	Scherzo SM-C18 (2 × 100 mm, 3 μm) (A) 1 mM formic acid and acetonitrile mixture (20:80, v/v) and (B) 10 mM formic acid solution containing 10 mM ammonium formate and acetonitrile mixture (80:20, v/v)	64–105	<11	Not determined	(92)
HPLC-ESI-TQ	6 Dialkyl phosphates: DEP, DETP, DEDTP, DMP, DMTP and DMDTP (0.02–0.5).	10.0 mL.	Lyophilization. Acetonitrile solubilization.	SB-C18 (4.6 mm, 1.8 μm).	93–107	0–8	Not determined	(93)
HPLC-ESI-TQ	6 Dialkyl phosphates: DEP, DETP, DEDTP, DMP, DMTP, and DMDTP (0.1).	0.6 mL	SPE with Strata X-AW	Luna HILIC (2.00 × 100 mm, 5 μm). (A) Water with 100 mM CH₃COONH₄ and (B) acetonitrile.	40–98	<22	<10	(94)
UPLC-ESI-TQ	Dialkyl phosphates: DEP, DETP, DEDTP, DMP, DMTP, and DMDTP (0.5)	4.0 mL.	LLE with diethyl ether and ethyl acetate using salting-out assisted extraction with MgSO_4_ and NaCl.	Hypersil GOLD HILIC column (2.1 mm, 100 mm, 3 μm) (A) Water with 10 mM CH₃COONH₄ and (B) acetonitrile with 5 of aqueous 10 mM CH₃COONH₄ solution.	82–117	3–20	Not determined	(95)
HPLC-ESI-TQ	5 Dialkyl phosphates: DEP, DETP, DMP, DMTP, and DMDTP (0.03–0.05)	2.0 mL.	VALLME with ethyl acetate and (NH4)2SO4.	Inspire C18 column (4.6 × 250 mm, 5 μm). (A) Water with 10 mM ammonium formate and (B) acetonitrile	85.0–114.1	<5	Not determined	(96)
UPLC-ESI-QTOF	6 Dialkyl phosphates: DEP, DETP, DEDTP, DMP, DMTP, and DMDTP (0.5–4.0).	0.3 mL	SPE with Strata-X-AW plates. Ion pair with tripropylammonium formate.	BEH C18 column (2.1 × 100 mm, 1.7 μm). (A) Water with 0.5 mM formic acid and tripropylammonium formate and (B) acetonitrile.	91–115	6–34, except for DMP that presented 34–70.	32–89	(97)
HPLC-ESI-TQ	Glyphosate and 6 DAPs (0.2–0.8)	0.2 mL.	Dilution by a factor of 2.	Dionex UTAC-LP2 ion chromatography column. (A) Water with 30 mM KOH and (B) water with 100 mM KOH.	85–100	1–18	Not shown	(98)
HPLC-ESI-TQ	9 Dialkyl and 5 monoalkyl phosphates (0.3–11).	3.0 mL Protein precipitation	–	Luna Phenyl–Hexyl column (2 × 150 mm, 3 μm) (A) Water and methanol (80:20 *v/v*) with 2.5 mM tributylamine and 2.5 mM acetic acid, and (B) methanol and water (95:5 *v/v*) with 2.5 mM tributylamine and 2.5 mM acetic acid	69–114	<20	Not determined	(99)
HPLC-ESI-TQ	6 DAPs 9 Organophosphate flame retardants. 1 Brominated flame retardant (0.5).	0.2 mL. Hydrolysis with β-glucuronidase.	SPE with Strata XAW	Hypersil GOLD aQ column (150 mm × 4.6 mm, 3 μm). (A) Water with 0.1% acetic acid and (B) acetonitrile and methanol (1:1 *v/v*).	89–118	2–11	0–9	(100)
HPLC-ESI-TQ	Organophosphate disulfoton and five metabolites: Disulfoton-sulfoxide, Disulfoton-sulfone, Demeton-S Demeton-S-sulfoxide, Demeton-S-sulfon (5.0)	0.5 mL.	QuEChERS with MgSO_4_ and CH_3_COONa. d-SPE with primary secondary amine, end-capped octadecylsilane, and MgSO_4_.	PCELL-PAK C18 MG II column (2.0 mm × 35 mm, 5 μm). (A) Water with 10 mM NH_4_HCO_2_ and (B) methanol	87–112	1–8	0–9	(101)
HPLC-APCI-TQ	4 Organophosphate pesticides 2 Metabolites of bisdithiocarbamate (0.004–0.01)	0.8 mL.	Lyophilization. Suspension with dichloromethane. Filtration.	Zorbax SB-C3 (4.6 × 150 mm, 5.0-μm). (A) Water with 0.1% formic acid and (B) methanol with 0.1% formic acid.	94–105	8–13	0–10	(102)
HPLC-ESI-TQ	3 Formamidine pesticides and 5 metabolites (1.0–2.0).	0.5 mL.	SLE with Chem Elut.	Atlantis T3 column (150 mm × 4.6 mm, 5 μm) (A) Water with 0.1% formic acid and (B) acetonitrile.	89–108	7–11	85–101	(103)
UPLC-ESI-TQ	9 Neocotinoid insecticides (0.1–0.3).	2.0 mL.	SPE with Oasis HLB.	YMC ODS-AQ C18 column (2.1 × 100 mm, 3 μm) Isocratic mode with water and acetonitrile containing 0.1% formic acid	81–103	<15	1–4	(104)
UPLC-ESI-TQ	6 Neonicotinoids and 1 metabolite (0.22–2.25).	1.5 g of Diaper. Liquid extraction with acetone.	SPE with Bond Elute PCX. SLE with ISOLUTE SLE+.	CAPCELLPAK C18 AQ (2.0 mm, 150 mm, 3 μm). (A) Water with 5 mM CH₃COONH₄ and 0.1% formic acid, and (B) acetonitrile	66–113	4–20	48–26	(105)
HPLC-ESI-TQ	6 Neonicotinoids and 2 metabolites (0.01–0.1)	0.2 mL. Enzymatic deconjugation with β-glucuronidase	Online SPE with Chromolith Flash RP-18e monolithic column (25 × 4.6).	Hypersil Gold aQ column (4.6 mm × 150 mm, 3 μm). (A) Water with 0.1% formic acid and (B) acetonitrile.	91–116	4–10	1–3	(106)
UPLC-ESI-TQ	6 Neonicotinoids and 4 metabolites (0.01–0.05)	0.5 mL.	SPE with Bond Elut Plexa	Kinetex phenyl/hexyl column (2.1 mm × 50 mm, 2.6 μm)	84–119	2–11	58–82	(107)
UPLC-ESI-TQ	6 Neonicotinoids and 4 metabolites (0.01–0.1)	3.0 mL. Hydrolysis with β-glucuronidase.	LLE with ethyl acetate	Zorbax SB-C18 column (2.1 × 100 mm, 3.5 μm). (A) Water with 0.1% formic acid and (B) acetonitrile.	71–107	3–17	1–2	(108)
UPLC-Orbitrap	7 Neonicotinoids insecticides and 1 metabolite: (0.2)	1.0 mL. Protein precipitation.	Online sample extraction with TurboFlow column Cyclone P (50 × 0.5 mm, 60 m particle size, 60 Å pore size).	Zorbax Eclipse Plus C18 column (100 × 2.1 mm, 1.8 μm). (A) Water with 0.1% formic acid and 4 mM NH_4_HCO_2_ and (B) methanol with 0.1% formic acid and 4 mM NH_4_HCO_2_.	78–116	<20	<20	(109)
UPLC-Orbitrap	6 Organophosphate and 3 pyrethroids metabolites (1–10)	0.5 mL.	On-column sample extraction with TurboFlow Cyclone P (50 × 0.5 mm, 60 m particle size, 60 Å pore size)	Zorbax Eclipse Plus C18 column (100 × 2.1 mm, 1.8 μm). (A) Water with 0.1% formic acid and 4 mM NH_4_HCO_2_ and (B) methanol with 0.1% formic acid and 4 mM NH_4_HCO_2_.	70–116	3–13	>20	(110)
HPLC-ESI-TQ	2 Organophosphate metabolites. 3 Pyrethroid metabolites 2 Phenoxyacetic acid metabolites. 1 Triazine metabolite. 1 DEET insect repellent (0.05–0.5)	1.0 mL. Enzymatic hydrolysis.	SPE with OASIS HLB.	ACE Excel 2 C18-PFP column (2.1 × 100 mm, 3.0 μm) (A) Water with 0.1% acetic acid in water and (B) acetonitrile.	90–118	<15	<10	(111)
UPLC-ESI-TQ	5 Organophosphate metabolites 5 Pyrethroid metabolites 2 Phenoxyacetic acid metabolites. <0.5 ng·mL^−1^	1 mL. Enzymatic hydrolysis.	SPE with OASIS HLB.	Betasil C18 column (2.1 mm, 100 mm, 3 μm). (A) Water with 5% methanol and 1% acetic acid and (B) acetonitrile.	90–110	4–25	<10	(112)
UPLC-ESI-TQ	3 Organophosphates and 5 metabolites. 5 Pyrethroids and 4 metabolites. 3 Neonicotinoids and 1 metabolite. 1 Pyrazol and 2 metabolites. 1 Triazole and 1 metabolite. (0.0001–0.02)	1.0 mL. Enzymatic hydrolysis.	SPE with Oasis HLB.	Kinetex XB-C18 column (2.1 × 50 mm, 1.7 μm) (A) Water with 5 mM amonium formate and (B) methanol with 5 mM ammonium formate.	71–114	<14	4–83	(113)
UPLC-ESI-TQ	6 Neonicotinoids. 7 Neonicotinoids. 5 Organophosphates. 2 Organophosphate. metabolites. 3 Carbamates. 1 Carbamate metabolite. 1 Phenylpyrazole. 3 Phenylpirazole metabolites. (0.20–1.39 ng·mL^−1^)	1 mL. Enzymatic hydrolysis.	Acetone and MgSO_4_ simplified extraction method.	Eclipse plus C18 column (2.1 mm × 100 mm, 1.8 μm). (A) Water with 0.1% formic acid and (B) acetonitrile.	49–134	6–18	2–51	(114)
UPLC-Orbitrap	18 Organophosphate metabolites 5 Pyrethroid metabolites 1 Carbamate metabolite 2 Phenoxyacetic acid metabolites 3 Chloroacetanilide metabolites (0.8–50)	5.0 mL. Enzymatic hydrolysis.	QuEChERS with EN salts (magnesium sulfate, sodium chloride, sodium citrate and disodium citrate sesquihydrate).	Hypersyl Gold C18 column (2.1 mm, 100 mm, 1.9 μm) (A) Water with 0.1% acetic acid, and (B) 0.1% acetic acid in methanol	40–127	1–28	10–59	(115)
UPLC-Orbitrap	2 Organophosphate. 1 Organophosphate metabolite. 1 Neonicotinoid. 1 Phenoxyacetic acid 1 Dithiocarbamate metabolite. 1 Triazine. 2 Benzimidazole. 1 Phenylurea. (1.7–20.4)	0.5 mL	QuEChERS using acetonitrile with 0.1% acetic acid, MgSO_4_ and NaCl.	Hypersil GOLD C18 (50 × 2.1 mm, 1.9 μm). (A) Water and (B) methanol	11–133	1–19	9–139	(116)
UPLC-ESI-TQ	4 Organophosphate 1 Neonicotinoid 2 Carbamate 1 Strobilurin 1 Carboxamide (0.58–39.0)	0.05 mL Protein precipitation.	–	Acquity ВЕН C18 Column (2.1 mm, 150 mm, 1.7 μm) (A) 5 mM NH_4_HCO_2_ in water and (B) MeOH.	80–115	2–11	<21	(117)
HPLC-ESI-QT	20 pesticides from different chemical classes (0.02–0.76)	3.0 mL.	DLLME with choline chloride and sesamol, including salting-out with NaCl.	X-Terra C18 (150 mm × 2.1 mm; 3.5 μm). (A) Water with 5 mM formic acid and (B) acetonitrile with 5 mM formic acid.	50–101	5–20	0–30	(118)
UPLC-ESI-TQ	87 pesticides from different chemical classes (0.011 to 3.494)	5.0 mL.	SPE with C18 Sep-Pak cartridges.	BEH C18 column (100 mm × 2.1 mm, 1.7 μm). (A) Water with 0.01% formic acid and, (B) methanol.	60–120	11–26	Not shown	(72)
UPLC-ESI-TQ	260 pesticides from different chemical classes (10)	100 μL	QuEChERS using MgSO_4_ and NaCl.	Kinetex C18 column (100 × 2.1 mm, 2.6 μm). (A) Water with 5 mM CH₃COONH₄ and 0.1% formic acid, (B) acetonitrile with 5 mM CH₃COONH₄ and 0.1% formic acid.	54.2–113.9	2.1–19.9	0–66	(119)
UPLC-Orbitrap	Retrospective semi-quantitative analysis of 263 pesticides from different chemical classes.	5.0 mL. Enzymatic hydrolysis.	QuEChERS with EN salts (magnesium sulfate, sodium chloride, sodium citrate and disodium citrate sesquihydrate)	Hypersyl Gold C18 column (2.1 mm, 100 mm, 1.9 μm). (A) Water with 0.1% acetic acid, and (B) methanol with 0.1% acetic acid.	–	–	–	(120)
UPLC-QqToF	Method 1: 15 Pesticides from different classes. Method 2: 25 Pesticides from different classes. Nicotine and Cotinine. (0.02 to 25)	20 mL Enzymatic hydrolysis and sulfatase.	Method 1: SPE with Strata-X-AW cartridge. Method 2: SPE with Strata-X cartridge.	HSS T3 column (2.1 mm, 150 mm, 1.8 μm). (A) Water with 0.01% formic acid, and (B) methanol with 0.01% formic acid.	84–124	<30	Not shown	(121)
HPLC-QqToF	12 Pesticides. 1 Pesticide metabolite. 7 Veterinary drugs. 5 Parabens. 1 UV filter. 1 Plastic additive. 2 Surfactants. 9 Additional substances. (4.3–113.2)	–	–	Positive mode: XSelect CSH column (2.1 × 100 mm; 3.5 μm). (A) Water with 0.1% formic acid and 10 mM NH_4_HCO_2_ and (B) acetonitrile with 0.1% formic acid and 10 mM NH_4_HCO_2_. Negative mode: Kinetex column (2.1 × 100 mm; 2.6 μm). (A) Water with 0.05% acetic acid and (B) acetonitrile with 0.05% acetic acid.	34–120	1–22	2–175	(122)
UPLC-ESI-TQ	2 Pesticides: -Aldicarb. -Carbofuran. 13 Drugs of abuse. 12 Benzodiazepines. 8 Antidepressants. 4 Anticonvulsants. 1 Analgesic. (0.5 and 50)	0.1 mL. Enzymatic hydrolysis. Protein precipitation.	–	Raptor Biphenyl column (50 mm × 3 mm, 2.7 μm). Method 1: For positive ESI, (A) 2 mM NH_4_HCO_2_ and 0.1% formic acid in water and (B) acetonitrile. Method 2: For negative ESI, (A) 0.2% acetic acid in water and (B) acetonitrile.	83–118	1–20	40–171	(123)
UPLC-ESI-TQ	18 Pesticides. 12 Personal care and consumer product. 5 Polycyclic aromatic hydrocarbons. 5 Organophosphate flame retardants. 5 Volatile organic compounds. 4 Tobacco alkaloids. 1 Drug of abuse. (0.1–0.01)	0.2 mL. Enzymatic hydrolysis.	SPE with Oasis HLB.	Method 1, including PCPs, PAH, and OPFR: Betasil C18 column (2.1 mm × 100 mm, 5 μm). (A) Water and (B) acetonitrile. Method 2, including VOC and pesticides. Hypersil Gold AQ column (3.0 mm × 100 mm, 3 μm). (A) Water with 0.1% acetic acid and (B) Methanol. Method 3, including tobacco and drugs of abuse. Synergi Polar RP column (2.0 mm × 100 mm, 2.5 μm). (A) Water with 0.1% acetic acid, and (B) acetonitrile with 0.1% acetic acid.	83–109	<20	<20	(124)
UPLC-ESI-TQ	31 Pesticides. 45 Plasticizers. 45 Phenols. (≤ 0.1 for 101 analytes and between 0.1 and 1.0 ng/mL for 18 analytes.)	0.5 mL. Enzymatic hydrolysis.	SPE with BS Elut NEXUS.	Method 1: Ultra AQ C18 column (2.1 mm × 100 mm, 3 μm). (A) 0.1% acetic acid in water and (B) 0.1% acetic acid in MeOH; Method 2: Betasil C18 column (2.1 mm, 100 mm, 5 μm) (A) Water and (B) ACN	80–120	0–11	16 with strong ME; 83 with moderate ME and 22 with soft ME^a^	(125)

a It is important to note that different approaches have been employed for determination accuracy, precision, and matrix effect. This table was elaborated for description purposes, please check the respective cited article for details.

The quantification of the hydrophilic active ingredient glyphosate, its derivative glufosinate, and the metabolite AMPA was accomplished using Ultra-High-Performance Liquid Chromatography-Electrospray-Triple Quadrupole (UPLC-ESI-TQ). This analytical approach involved derivatization with acetate/acetic anhydride and trimethyl orthoacetate. By integrating methylation and acetylation reactions, this method circumvented extraction steps, resulting in a streamlined sample preparation process (81).

Furthermore, the viability of derivatization with FMOC-Cl for determining glyphosate and AMPA was investigated, with testing conducted on 20 farmers. Notably, one participant consistently recorded a glyphosate value of 200 ng·L^−1^ on a spraying day (82). In another method for glyphosate and AMPA determination, high-resolution mass spectrometry (UPLC-Orbitrap) coupled with cold-induced phase separation and hydrophilic DPX was employed with interesting results (83).

An investigation into the utilization of a microbore hypercarb column, comprised of porous graphitic carbon, was also conducted for determining glyphosate and three of its metabolites. While this method was specifically applied for analyses in pig urine to monitor contamination through feed, its potential extends to broader biomonitoring applications (84).

In another study, a method was developed employing protein precipitation for glyphosate, glufosinate, diquat, and paraquat, aiming at urine and blood samples for forensics analyses using a C18 reversed phase column, which was designed to allow the alkyl phase to remain accessible in highly aqueous mobile phases (85). Conversely, for enhanced chromatographic separations, a method targeting the quaternary ammonium chlormequat was developed employing HILIC and SPE with Isolute HCX-Q, yielding satisfactory results (86).

The fungicide mancozeb, another hydrophilic active ingredient pertinent to biomonitoring studies, is notable for its primary evaluated metabolite, ethylenethiourea (ETU), which was determined through supported liquid extraction using diatomaceous earth, followed by High-Performance Liquid Chromatography-Atmospheric Pressure Chemical Ionization-Single Quadrupole (HPLC-APCI-SQ). In a study encompassing 261 individuals from the UK general population, a detection frequency of 46% was observed (87).

In a separate study focusing on ETU biomonitoring, alkaline hydrolysis and subsequent on-column extraction was employed with determination by HPLC-APCI-TQ. During dermal exposure experiments, 10% of the administered dose was detected in urine (88). Moreover, in the quest for biomarkers of pesticide exposure, a method for determining 5-hydroxythiabendazole was elaborated, aiming to assess exposure to the benzimidazole fungicide thiabendazole (89).

Regarding phthalimides fungicides like folpet and captan, an HPLC-APCI-TQ method for determining metabolites was developed, utilizing Solid Phase Extraction (SPE) with Oasis HLB cartridges. Four workers were evaluated during 5 days of occupational exposure, with concentrations ranging between LOD and 8.5 ng·mL^−1^ (90). Additionally, an HPLC-APCI-TQ method, coupled with SPE using the cation exchange cartridge Strata X-C, was employed to determine 7 atrazine metabolites, utilized in the USA National Health and Nutrition Examination Study (91).

Recent advancements in LC–MS/MS have facilitated the development of multi-residue methods for detecting DAPs without requiring derivatization, thereby streamlining sample preparation efforts. A method for determining DEP, DETP, DMP, and DMTP by UPLC-ESI-TQ employed two sequential SPE procedures with Oasis WAX cartridge and a C18 column. This method was utilized for biomonitoring 225 three-year-old children, with detection frequencies ranging from 80 to 100% and medians between 0.6 and 14.4 ng·mL^−1^ (92).

Another approach was also devised to simplify sample preparation for the determination of 6 DAPs. This method involved lyophilization followed by dissolution in acetonitrile. In a study conducted with 30 children from Hyderabad, India, the average concentration of these metabolites ranged from 0.06 to 12 ng·mL^−1^, suggesting widespread exposure to pesticides (93).

In a different investigation, HILIC mode was selected to achieve effective chromatographic separation of DAPs, prompting exploration of various methodologies. The determination was performed using SPE with Strata X-AW for extraction, employing a Luna HILIC column (94). Alternatively, LLE with diethyl ether and ethyl acetate, alongside salting-out assisted extraction with MgSO_4_ and NaCl, also adopting HILIC chromatography but with a Hypersil Gold HILIC column, was assessed for the same 6 DAPs (95). In a separate investigation, five DAPs were determined using VALLME with ethyl acetate and (NH_4_)_2_SO_4_, however employing an Inspire C18 column for separation (96).

Ion-pair chromatography employing tripropylammonium formate emerged as a promising approach for the determination of DAPs. This method involved SPE utilizing Strata-X-AW coupled with UPLC-ESI-Quadrupole Time-of-Flight Mass Spectrometry (QTOF). When applied to biomonitoring the Norwegian mother–child cohort, consisting of approximately 100 participants, the method yielded detection rates of 40% for DMP, 95% for DEP, 96% for DMTP, 50% for DETP, 15% for DMDTP, and 1% for DEDTP (97).

A more comprehensive biomonitoring approach for organophosphorus compounds involved 9 DAPs and 5 monoalkylphosphates, utilizing ion pair chromatography with tributylamine. Accessing 19 volunteers, the concentration of these compounds totaled 20 ng·mL^−1^ (99). Ion chromatography was also employed for separation of six DAPs and glyphosate, and the analysis of individuals adhering to an organic diet demonstrated an 80% detection frequency, while 78% detection was observed in 40 subjects with suspected glyphosate exposure (98).

Several methods have been developed for biomonitoring organophosphorus compounds derived from additional sources. One such method identified 6 DAPs, 9 organophosphate flame retardants, and 1 brominated flame retardant. Concentrations were found to be higher in a cohort of 145 firefighters compared to 158 individuals from the general population, ranging between 2 and 37 times greater, encompassing the primary active ingredients along with specific exposure biomarkers (100). As also observed in another work that quantified disulfoton and five of its metabolites, employing QuEChERS and d-SPE techniques (101).

Expanding the scope, an HPLC-APCI-TQ approach was employed to ascertain 4 organophosphate pesticides (acephate, methamidophos, dimethoate, and omethoate) alongside 2 metabolites of bisdithiocarbamate fungicides (ETU and 1-phenyl 2-thiourea). This method encompassed lyophilization, dichloromethane suspension, filtration, and acetonitrile resuspension (102). Furthermore, a methodology was introduced for the determination of formamidine pesticides (amitraz, chlordimeform, formetanate) and five of their metabolites, underscoring the necessity for robust methodologies concerning emerging active ingredients in biomonitoring studies (103).

Given the widespread indoor use of neonicotinoid insecticides, it is imperative to assess their presence in the general population, especially among infants. Consequently, a method was developed for the simultaneous determination of 9 parent compounds. Then, 10 children were analyzed, revealing detection frequencies ranging from 0 to 80% (104).

Various studies have delved into different life stages, including early childhood, to understand pesticide exposure in an expossome approach. One such method involved the determination of 6 neonicotinoid insecticides and one metabolite through solvent extraction of urine from diapers, followed by SPE and SLE. This approach, applied to a cohort of 50 diapered children, revealed detection rates of 78% for *N*-desmethylacetamiprid and 84% for dinotefuran (105).

In another study focusing on neonicotinoids, researchers employed an enzymatic hydrolysis technique coupled with online SPE to detect 6 neonicotinoids and 2 metabolites. Analyzing samples from 60 individuals from the general population, it was found that 95% were contaminated with 3-diethyl-carbamoyl benzoic acid and 83% with 3-ethyl-carbamoyl benzoic acid (106).

Optimization of extraction methods was explored using different SPE cartridges to determine 7 neonicotinoid insecticides and 4 metabolites. Bond Elut Plexa emerged as the preferred choice, with at least two analytes detected in each sample from an analysis of 20 healthy volunteers (107). Employing a different extraction strategy, a method utilizing LLE with ethyl acetate was developed for the quantification of 6 neonicotinoids and 4 metabolites, highlighting the importance of exploring diverse extraction techniques (108).

Evaluation of UPLC-Orbitrap with online sample preparation using TurboFlow was conducted for the determination of 7 neonicotinoid insecticides and 1 metabolite, leading to the development of a semi-automated method. This approach showcased the potential of such strategies for biomonitoring studies (109). UPLC-Orbitrap with Turboflow was also employed to determine 6 organophosphates and 3 pyrethroid metabolites, which was applied to 30 individuals revealing the detection of 4-nitrophenol, 2-diethylamino-6-methylpyrimidin-4-ol, and 2-isopropyl-6-methyl-4-pyrimidinol in 6 samples, alongside the detection of 11 other pesticides through untargeted analyses (110).

In a broader scope for pesticide exposure assessment using HPLC-ESI-TQ setup, a method was proposed for the determination of 9 metabolites of organophosphates, pyrethroids, phenoxyacetic acids, and DEET. This method employed enzymatic hydrolysis and SPE using Oasis HLB. The analysis of 101 samples from a cohort study revealed detection frequencies of 98% for 3-PBA, 91% for IMPY, 89% for TCP, 66% for 2,4-D, 11% for F-3-PBA, and 0% for 2,4,5-T (111).

Moreover, a parallel investigation employing a similar strategy developed a method to determine 12 metabolites from organophosphates, pyrethroids, and phenoxyacetic acids, utilizing enzymatic hydrolysis and semi-automated SPE with Oasis HLB (112). Furthermore, another study, employing enzymatic hydrolysis with Oasis HLB, scrutinized 13 pesticides and 13 metabolites, encompassing organophosphates, pyrethroids, neonicotinoids, a phenylpyrazol, and a triazole (113).

A wider HPLC-ESI-TQ method targeting 28 compounds including neonicotinoids, organophosphates, carbamates, a pyrazole, and their metabolites, was developed. It incorporated enzymatic hydrolysis followed by a simplified extraction with acetone and MgSO_4_, revealing ubiquitous exposure to the evaluated pesticides in the form of metabolites among 20 children in South China (114).

UPLC-Orbitrap analysis was also explored for the determination of 29 urinary metabolites from various chemical and use classes, offering the advantage of post-target retrospective identification of biomarkers (115). In addition, another UPLC-Orbitrap method examined 10 nitrosable pesticides using different extraction methods; protein precipitation, SPE, and QuEChERS, showcasing promising potential for future biomonitoring studies (116).

An investigation about sample preparation evaluated protein precipitation, SPE with C18, QuEChERS, and d-SPE with PSA ahead of UPLC-ESI-TQ analysis. In conclusion, protein precipitation emerged as the most efficient strategy for determining 9 active ingredients in a low sample volume of 50 μL, ideal for postmortem analyses and biomonitoring studies with limited sample availability (117).

Furthermore, a comparison of extraction methods for 20 pesticides spanning 12 different chemical classes was conducted. This study revealed recovery rates of 79, 70, and 63% for SPE, DLLME with choline chloride and sesamol, and DLLME with chloroform and acetonitrile, respectively. Although DLLME showed promise in terms of time and solvent use, SPE demonstrated superior extraction efficiency (118).

In a more complete analytical strategy, a complementary approach employing both GC–MS/MS and LC–MS/MS was developed, utilizing unified sample preparation using SPE with C18 Sep-Pak cartridges. Combined, a total of 205 pesticides were assessed (72). Also focusing on robust and extensive multi-residue methods, a very interesting UPLC-ESI-TQ method targeting 260 pesticides was introduced, addressing biomonitoring, clinical, and forensic studies (119).

In an alternative strategy, a retrospective semi-quantitative analysis with 263 theoretical pesticide metabolites was performed using UPLC-Orbitrap. A total of 26 compounds were identified in actual samples. These compounds presented a 4–18% detection frequency with estimated concentrations of 1.2–14.7 ng·mL^−1^, except for propachlor oxanilic acid, which reached 141 ng·mL^−1^ (120).

The evaluation of various pesticide residues and other contaminants can be performed using TOF detection. For instance, two distinct UPLC-QTOF methods were employed to detect 38 pesticide exposure biomarkers, in addition to nicotine and cotinine. A subsequent analysis of samples from 15 pregnant women unveiled that most of the identified compounds were metabolites rather than the primary active ingredient (121).

In a study utilizing HPLC-QTOF, 38 compounds were identified in urine samples without the need for sample preparation and extraction. These compounds included 12 pesticides from different chemical classes, 1 pesticide metabolite, 7 veterinary drugs, 5 parabens, 1 UV filter, 1 plastic additive, 2 surfactants, and 9 additional substances, aiming to provide a rapid and straightforward method for high throughput screening (122).

Broad urine analyses aimed at unspecific toxicological emergencies were also developed via UPLC-ESI-TQ. This method facilitates the identification and quantification of 40 different compounds, including analgesics, benzodiazepines, antidepressants, anticonvulsants, drugs of abuse, and pesticides like aldicarb and carbofuran. Therefore, this approach enables rapid identification of toxic substances in cases of poisoning (123).

Multi-class analyses represent an exciting and cost-effective strategy. One of these methods successfully determined 50 analytes, including pesticides and compounds from different applications, using SPE with OASIS HLB followed by three injections on UPLC-ESI-TQ utilizing different columns (Betasil C18, Hypersil Gold AQ, and Synergi Polar RP), highlighting the potential of this reported approach (124).

Another biomonitoring method targeting various environmental pollutants was developed with a particular emphasis on pesticides. A total of 121 chemicals were addressed, including 45 plasticizers, 45 phenols, and 31 pesticides. The optimized method was validated using standard reference materials and proficiency test urine samples before analyzing 21 samples (125).

Methods employing LC–MS/MS as a determination technique were initially developed for singular or a few active ingredients and metabolites. Subsequently, multi-residue approaches for analytes from the same chemical group were introduced (119, 120). In recent years, strategies for determining pesticides from several classes have been assessed, and in some cases, contaminants from consumer products have been evaluated in the same analytical run, independently of employing Triple Quadrupole, Time of Flight, or Orbitrap spectrometers (120–125).

Unlocking the true potential of chromatography-mass spectrometry enables the analysis of numerous compounds of interest in high throughput (126). These advancements were made possible due to the expanded range of analytes at the LC inlet compared to the GC, which necessitates thermal stability (127). Furthermore, novel mass spectrometry ionization methods and direct sampling techniques have broadened the scope of applications, with the potential for further expansion (128).

## 4 Applications

**Figure 3:**
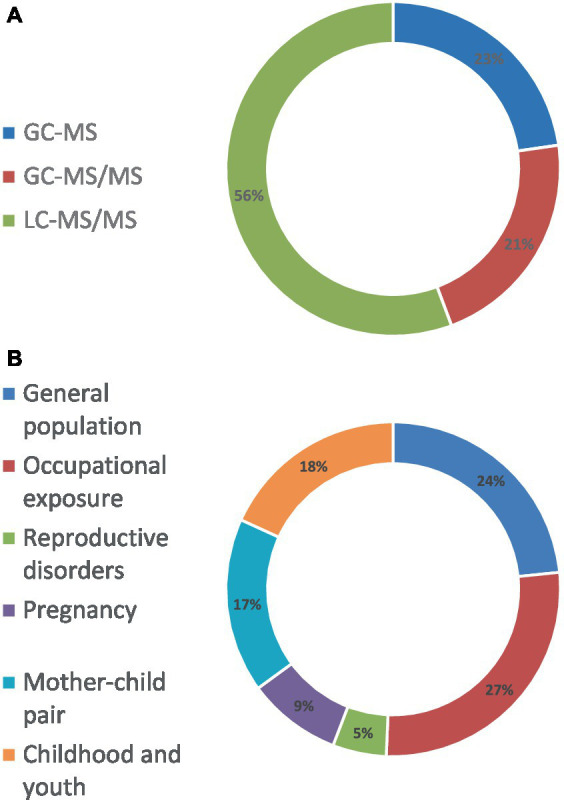
Distribution of literature analyzed based on the method of determination utilized **(A)** and the subject of the study **(B)**.

Chromatography-mass spectrometry equipment has been utilized for the analysis of pesticide residues in urine samples, aimed at assessing potential health effects within a variety of population groups (Table 4 and Figure 3). These investigations are typically categorized into three main groups: assessments of exposure within the general population, evaluations of occupational exposure, and examinations focused on early stages of life.

**Table 4:** Summary of the approached applications for pesticide determination in urine.

Analytes	Number of participants	Method description	Study objective	Conclusion	References
Permethrin.	6 Healthy volunteers.	HPLC-MS/MS.	Determine the pharmacokinetics of dermal exposure to permethrin when employing this insecticide as repellent in clothes.	3-Phenoxybenzyl alcohol glucuronide was the main metabolite, but the most part of radioactivity was present in polar fractions composed of unknown metabolites.	(129)
Pesticides: 4 Organophosphate metabolites. 1 Pyrethroid metabolite. 1 Phenylpyrazole and 1 metabolite. 1 Pyridinecarboxamide. 16 Phthalates 4 Bisphenols	16 Volunteers followed for 6 months.	Enzymatic hydrolysis. SPE with Oasis HLB. UPLC-ESI-MS/MS.	Assess the framework of using urine and hair analysis together, with repeated sampling over time for biomonitoring endocrine disruptors.	Most biomarkers were detected in higher frequency in urine than in hair. However, the results were more constant in hair.	(130)
1 Aminophosphonic acid 5 Pyrethroids 7 Organophosphate metabolites. And others contaminants.	15 Participants of general population.	Glyphosate: Derivatization with FMOC. LLE. UPLC-ESI-TQ Pyrethroids and Chlorpyrifos -Derivatization with MTBSTFA. LLE. GC–MS/MS. DAPs Derivatization with chloroiodopropane. SPE with Oasis WAX cartridges. GC–MS/MS.	Assess the environmental contamination of Kinshasa (Democratic Republic of Congo) population in a pilot study.	The approached population is exposed to a wide variety of pesticides and other environmental pollutants.	(131)
Glyphosate	50 Adults.	SPE with Strata SAX cartridges. HPLC-ESI-MS/MS (132).	Explore the glyphosate exposure of general population from Ireland.	Glyphosate was detected in 20% of participants, even with a relatively high detection limit of 0.5 ng·mL^−1^.	(133)
Glyphosate. AMPA.	46 First round. 33 Second round. Participants of general population.	First round: Protein precipitation with acetonitrile. Derivatization with 2,2,2-trifluoroethanol. GC–MS/MS. Second round: Acidification with formic acid. HPLC-ESI-TQ.	Explore the glyphosate exposure of Portuguese adults.	Exposure to glyphosate was detected in the participants at both rounds.	(134)
7 Neonicotinoids and 3 metabolites	75 Participants from general population, age 13–80.	SPE with Presep RPP cartridge and ENVI-Carb/PSA cartridge. HPLC-ESI-MS/MS.	Biomonitoring neonicotinoid pesticides in Kumasi (Ghana).	The most part of the participants was exposed to multiple neonicotinoid insecticides.	(135)
3-PBA	50 Adults from general population.	Enzymatic hydrolysis. SPE with Oasis HLB cartridge. HPLC-ESI-MS/MS.	Assess pyrethroids exposure by 3-PBA concentration in urine employing samples of weeks 1, 2, and 6 of a six-week monitoring period in adults from North Carolina (USA).	It was indicated that a single measure of urinary 3-PBA may not be sufficient to characterize average exposure.	(136)
3-PBA	50 Adults from general population.	Enzymatic hydrolysis. SPE with Oasis HLB cartridge. HPLC-ESI-MS/MS.	Assess pyrethroids exposure by 3-PBA concentration in urine employing 24 h sampling in adults from North Carolina (USA)	Exposure to pyrethroids was positively associated with time spent outside, consumption of coffee and breads, and creatinine levels.	(137)
4 Pyrethroids metabolites	306 Young men living in urban area.	Acid hydrolysis with HCl LLE Derivatization by HFIP and DIC. GC–MS	Assess body burden of pyrethroids in young men of Łódź (Poland) and identify predictors of pyrethroid exposure.	Non-dietary factors, especially dog ownership and pesticide use on household pets contributed significantly. Moreover, seeds and nuts consumption and juice intake were significant.	(138)
2,4-D. TCP. 3-PBA. Pentachlorophenol	127 Adult caregivers of children.	Acid hydrolysis with HCl. LLE with dichloromethane. Derivatization with MTBSTFA. GC–MS.	Quantify urinary levels of pesticides from different classes and associate them with sociodemographic characteristic and lifestyle factors.	Specific sociodemographic characteristics and lifestyle factors were associated with increased exposure to different pesticides.	(139)
1 Phenoxyacetic acid metabolite. 3 Organophosphate metabolites. 3 Pyrethroids metabolites. 6 Neonicotinoid metabolite. 2 Diethyltoluamide repellent metabolite.	30 People. 30 Dogs.	SPE. HPLC-ESI-TQ.	Examine pesticide exposures of people and their dogs from North Caroline and New Jersey (USA). Moreover, validate the use of silicone wristbands as alternative method	This type of study can identify health risks to humans and pets.	(140)
6 General metabolites of organophosphates. 4 Specific metabolites of organophosphates. 4 Pyrethroids metabolites. 2 Phenoxyacid herbicides. 1 Diethyltoluamide repellent and 2 metabolite.	100 Participants of general population.	SPE HPLC-MS/MS	Biomonitoring Australian residents (Queensland) using a cost-effective pooled urine-sampling approach.	A “U-shaped” trend for five organophosphate metabolites was observed with highest exposure in the youngest and oldest age strata.	(141)
4 DAPS; DMP, DMTP, DEP, DETP.	240 participants from general population	Acid -Hydrolysis with HCl. SPE with Bond Elut PPL. Derivatization with PFB-Br GC–MS/MS	Estimate exposure to organophosphate pesticides considering dietary habits and organic food consumption.	The employed estimation was consistent with the urinary concentration of organophosphate metabolites.	(142)
6 DAPS	4,446 participants from general population	Enzymatic hydrolysis Derivatization with PFB-Br. LLE. GC–MS	Assess the effects of exposure to organophosphates on lung function.	Exposure to organophosphates was associated with detrimental effects on lung function.	(143)
4 Specific organophosphate metabolites 2 Phenoxyacetic acids 4 Pyrethroid metabolites	322 Participants from general population.	Enzymatic hydrolysis. SPE with Oasis HLB. HPLC-ESI-MS/MS.	Evaluate the exposure of the general population from eight countries; USA, Greece, China, India, Saudi Arabia, Japan, Korea, and Vietnam.	The overall distribution of pesticides concentration in urine was similar. Also, worldwide exposure to pesticides was indicated.	(144)
3-PBA	2,116 Participants of general population.	Enzymatic hydrolysis. SPE with OASIS HLB. HPLC-APCI-MS/MS.	Assess associations between mortality and cause-specific mortality with exposure to pyrethroids in the United States population.	Exposure to pyrethroid insecticides was significantly associated with increased risk of death and cardiovascular disease mortality.	(145)
4 Neonicotinoids and 2 metabolites	3,038 Participants of general population.	Enzymatic deconjugation with β-glucuronidase. Online SPE with Chromolith Flash RP-18e. HPLC-ESI-MS/MS.	Evaluate neonicotinoids exposure in the United States population.	The frequency detection of at least one neonicotinoid compound was 49%.	(146)
1 Pesticide, pyrimethanil. 1 Polycyclic aromatic hydrocarbon, pyrene. 1 Ultraviolet-light absorber, oxybenzone.	432 Participants of general population.	Enzymatic hydrolysis. UPLC-ESI-MS/MS.	Evaluate if the loss of function of the filaggrin protein from the skin barrier due to polymorphism promote increased dermal exposure in Lund and Malmö (Sweden).	FLG genotype influences the dermal absorption.	(147)
12 Metals and metalloids. 2 Mycotoxins. 12 Pesticide metabolites; 1 Organophosphate. 4 Pyrethroids. 2 Phenoxyacetic acids. 1 Dithiocarbamate. 1 Aminophosphonic acid. 1 Benzimidazole. 1 Triazole. 1 Aminopyrimidines	350 Young adults from a rural community.	Different LC–MS/MS methods were employed.	Characterize the associations between urinary concentrations of pesticides, metals, and mycotoxins and decline in kidney function in the population from northwest Nicaragua.	Pesticides, metals, metalloids, and mycotoxins were not significantly associated with kidney function loss.	(148)
1 Sulfanylylcarbamate. 1 Phenylcarbamate. 1 Imidazole. 1 Triazole.	16 Members of farmer families. 38 Members of non-farmers families from agricultural communities.	Each pesticide was analyzed by as specific method using LC–MS/MS.	Compare pesticide exposure of farmer and non-farmer families in agricultural communities in the North-West of Netherlands.	Only carbendazim presented association between concentrations in hand wipes and metabolites in urine, showing high dermal exposure.	(149)
Penconazole and 2 metabolites.	22 Pesticide applicators	Enzymatic hydrolysis. HPLC-ESI-MS/MS.	Evaluate exposure to penconazole and the association with dermal exposure.	Hydroxy-penconazole exposure was positively associated with actual body exposure and actual total exposure. Showing that this is an interesting biomarker for biomonitoring.	(150)
ETU (Mancozeb metabolite)	29 Male agricultural workers, which apply pesticide using tractors.	Acid hydrolysis with HCl. LLE with diethyl ether. GC–MS/MS.	Evaluate the exposure determinants to mancozeb for mechanized applicators that employed tractors in Italian vineyards at Mantova and Pavia province (Italy)	Main exposure determinants were the type of tractor cabin (open or closed) and Personal Protective Equipment (coverall and gloves).	(151)
ETU (Mancozeb metabolite)	29 Male agricultural workers, which apply pesticide using tractors for 38 workdays.	Acid hydrolysis. LLE with diethyl ether. GC–MS/MS.	Estimate the mancozeb dose absorbed by pesticide applicators in a working day and compare with the Fixed Fractional Approach.	Estimation of the absorbed dose considering real duration of exposure can result in a higher correlation with ETU concentration in urine.	(152)
ETU (Mancozeb metabolite)	16 Pesticide applicators	Supported liquid extraction with diatomaceous earth column ChemElut. UPLC-ESI-MS/MS.	Propose a procedure to establish a biological exposure limit using real-life data and occupational exposure limits for mancozeb	A procedure was presented with focus on the determination of biological exposure limits for pesticides, in this case, mancozeb.	(153)
Imidacloprid 6-chloronicotinic acid	43 Pesticide applicators	Enzymatic hydrolysis. SPE with cation exchange Polar Enhanced Polymer. UPLC-ESI-MS/MS.	Provide reference of health risk evaluation due to imidacloprid exposure.	The estimated absorbed daily dose was 0.52–248.05 μg·kg^−1^·d^−1^ for imidacloprid.	(154)
Glyphosate. AMPA.	18 Dairy farmers applicators. 17 Non-applicators.	LC–MS/MS.	Determine glyphosate historical exposure in 1997 and 1998 in farmers that self-reported exposure at Wisconsin (United States).	Glyphosate was not detected in any non-applicator. Whereas 39% detection frequency was determined in applicators.	(155)
Glyphosate	59 Pesticide sprayers.	HPLC-MS/MS.	Determine glyphosate toxicokinetics among farmers at Long District (Thailand).	The mean urinary elimination half-life for the one-time and two-time sprayer group were 7.0 and 18.1 h.	(156)
Glyphosate	180 Farmers	Dilute-and-shoot. UPLC-MS/MS.	Investigate glyphosate effects on oxidative stress, inflammation, and lung function after application.	Glyphosate exposure promoted a significant effect on oxidative stress and lung function in farmers.	(157)
TCP (Chlorpyrifos and methyl chlorpyrifos metabolite)	28 Farmers. 43 Non-farmers from agricultural communities.	Enzymatic hydrolysis. SPE with OASIS HLB plate. UPLC-APCI-TQ.	Perform a longitudinal evaluation of chlorpyrifos and chlorpyrifos-methyl exposure in agricultural workers in South Tyrol (Italy).	Non-farmers showed higher levels than farmers in the non-application season, suggesting the existence of unknown sources of exposure.	(158)
4 General metabolites of organophosphates: DMP, DEP, DETP, and DEDTP.	80 Pesticide sprayers. 90 General population.	Acid hydrolysis with HCl. LLE with diethyl ether and acetonitrile. Derivatization with PFB. GC–MS (159)	Determine associations between diseases and occupational exposure of pesticide applicators from central Greece.	DETP was significantly associated with increased cases of allergic rhinitis.	(160)
6 Specific metabolites of organophosphate pesticides. 2 General metabolites of pyrethroid pesticides.	48 Farmers. 77 Urban population.	Enzymatic deconjugation. SPE with OASIS HLB cartridges. HPLC-ESI-TQ.	Compare the exposure of general population and farmworkers of Catalonia and Galicia (Spain) to organophosphate and pyrethroid pesticides.	The exposure of farmworkers was twice the exposure of the general population of urban environment.	(161)
6 General metabolites of organophosphates	79 Pesticide sprayers	GC–MS	Compare urinary residues of pre and post pesticide application with cognitive performance.	The scores obtained in cognitive tests were significantly lower after application season, but no correlations were observed for urinary concentrations of DAPs. Personal protective equipment reduced exposure.	(162)
6 General metabolites of organophosphates	160 Huichol farmers. 30 Huichol non-farmers.	Derivatization with PFB. LLE with hexane and dichloromethane. GC–MS/MS (163).	Assess the methylation profiles of the CDKN2A and CDKN2B genes in the indigenous Huichol farmers.	Occupational exposure to organophosphate pesticides can modify the methylation of the CDKN2B gene.	(164)
6 General metabolites of organophosphates	117 Flower growers.	Acid hydrolysis with HCl. LLE with diethylether/acetonitrile. Derivatization with PFB. LLE with hexane Clean-up with three-layer column of 0.3 g of Florisil, 0.1 g of Bondesil-PSA. GC–MS.	Evaluate occupational exposure related to urinary concentration of general metabolites of organophosphate pesticides in flower growers from Mexico.	Greenhouse workers were more exposed than outdoor flower growers. Additionally, the lack of use of personal protective equipment increased contamination.	(165)
56 Pesticides and 14 metabolites	42 Florists 42 General population	Extraction with ethyl acetate. LC-ESI-MS/MS.	Evaluate occupational health risks of Belgian florists due to pesticide exposure.	Florist were occupationally exposed to a variety of pesticides.	(166)
4 Organophosphate general metabolites: DMP, DMTP, DEP, and DETP,	230 Pest control workers.	Acid hydrolysis with HCl. LLE with diethylether–acetonitrile. Derivatization with PFB-Br. Clean-up with column with Florisil, PSA, and sodium sulfate anhydrous. GC–MS.	Assess the impact of Paraoxonase 1 gene polymorphisms and the OP metabolites in the urine of pest control workers from Nagoya (Japan).	Metabolite concentrations in urine were not significantly associated with PON1 polymorphisms. Negative associations were significant between DAP concentrations and activities of fenitrotion oxonase and arylesterase.	(167)
Glyphosate Fluroxypyr	19 Amenity horticulturists	SPE with Strata SAX cartridges. HPLC-ESI-MS/MS.	Evaluate occupational exposure of amenity horticulturists.	The determined urinary levels were comparable to those reported for agricultural workers.	(132)
Glyphosate	20 Amenity horticulturists	SPE with Strata SAX cartridges. HPLC-ESI-MS/MS.	Assess occupational exposure of amenity horticulturists employing multiple spot sampling of urine.	The highest concentrations of glyphosate were determined in samples obtained 3 h after exposure task.	(168)
Pyrimethanil and its metabolite 4-hydroxypyrimethanil.	2 Healthy volunteers. 413 Participants from general population. 18 Horticulturists.	Enzymatic hydrolysis. SPE with Isolute. HPLC-ESI-MS/MS.	Determine pyrimethanil exposure in general and occupationally exposed populations from southern Sweden.	After biomarkers determination in controlled exposure experiments, 4-hydroxypyrimethanil was detected in 48% of the general population and in 96% of the occupationally exposed horticulturists.	(169)
7 Organochlorine pesticides. 7 PCBs congeners	111 Infertile men, resulting in 45 hair, 96 urine and 70 serum samples.	SPME. GC–MS.	Evaluate PCBs, DDTs and HCB exposure of infertile and healthy fertile men by hair, urine and serum samples at Punjab and Khyber Pakhtunkhwa (Pakistan).	The levels of pollutants were higher in infertile men with few exceptions. HCB was negatively correlated with sperm motility.	(170)
6 General organophosphate metabolites: DMP, DMTP, DMDTP, DEP, DETP, and DEDTP.	159 Men.	Acid hydrolysis with HCl. LLE with acetonitrile and diethylether. Derivatization with PFB-Br. GC–MS.	Assess the environmental exposure to organophosphate pesticides and their association with frequency of disomy among adult men.	Disomy rates were significantly associated with specific organophosphate metabolites.	(171)
11 Pesticides, metabolites of organophosphates and pyrethroids, and phenoxyacetic acids.	594 Reproductive-age women.	Enzymatic hydrolysis. SPE with OASIS HLB. HPLC-ESI-MS/MS.	Examine association between pesticides and endometriosis in reproductive-age women from United States.	Exposure to diazinon and chlorpyrifos may be associated with endometriosis.	(172)
9 metabolites: -6 General organophosphates metabolites. -3 Pyrethroids metabolites.	615 Women	GC–MS/MS	Assess the effects of organophosphate and pyrethroid exposure over time to pregnancy and infertility at China.	Exposure of organophosphate and pyrethroid pesticides were associated with decreased fertility.	(173)
6 General organophosphates metabolites.	522 Women	GC–MS/MS	Evaluate birth outcomes with DAPs concentrations at preconception at *in vitro* fertilization	Organophosphate exposure was associated with reduced successful implantation, clinical pregnancy, and live birth. No association were identified for total and mature oocyte counts, best embryo quality, fertilization, E_2_ trigger levels, and endometrial wall thickness	(174)
6 General organophosphate metabolites	62 Pregnant women.	SPE. HPLC-ESI-MS/MS.	Assess intra-individual variations of DAPs and reproducibility in the determination of organophosphate exposure in Japanese pregnant women.	Sampling of urine in the afternoon correlated better than first morning void for biomonitoring, showing that this type of sample can be important.	(175)
TCP, organophosphate chlorpyrifos specific metabolite.	50 Pregnant farmers.	Enzymatic hydrolysis. Online SPE. HPLC-MS/MS.	Identify biological alterations associated with chlorpyrifos exposure among pregnant farmers in Thailand.	The associated alterations were related to oxidative stress, cellular damage and repair, and systemic inflammation.	(176)
4 General metabolites of organophosphates. 4 Specific metabolites of organophosphates.	573 Pregnant women.	Enzymatic hydrolysis. QuEChERS extraction. UPLC-ESI-MS/MS.	Evaluate the exposure factors of pregnant women to organophosphate pesticides in Spain.	Exposure to organophosphate pesticides was associated with intake of fruits and vegetables, body mass index before pregnancy and smoking habit during pregnancy.	(177)
3-FBA. 4F-3-FBA. DBCA.	480 Pregnant women from non-rural areas.	LLE with ethyl acetate. UPLC-ESI-MS/MS.	Assess the prenatal exposure to pyrethroids of women from non-rural areas.	Pyrethroid exposure was positively associated with consumption of bananas and oranges, and the number of fruits types the women regularly ate. Also, exposure was negatively associated with early pregnancy body mass index, unemployment, frequent intake of apples, and washing fruits and vegetables with soda or hot water.	(178)
6 General organophosphate metabolites. 5 Pyrethroids metabolites.	30 Pregnant women	Acid hydrolysis with HCl. LLE with n-hexane. Derivatization with MTBSTFA. GC–MS/MS.	Find association between parent insecticides in blood and their metabolites in urine in pregnant women from Taiwan.	The most detected pyrethroids were present in domestic products. Therefore, exposure may have occurred mostly using domestic insecticides.	(179)
10 phenols, including 2 metabolites of phenoxyacids herbicides. 7 parabens. 16 phthalate metabolites	200 Pregnant women	Different LC–MS/MS methods were employed.	Describe the exposure of pregnant women from Odense (Denmark) to phthalates, parabens and phenols.	Detectable levels of phthalate metabolites, parabens and phenols were observed in almost all pregnant women.	(180)
4 General metabolites of organophosphates. 7 Phenols. 10 Phthalate metabolites.	152 Pregnant women	Different LC–MS/MS methods were employed.	Evaluated associations to exposure to non-persistent chemicals of pregnant women at the second and third trimester employing repeated urinary sampling. Participants were from Barcelona (Spain), Grenoble (France), and Oslo (Norway).	It was suggested that higher exposure to phthalates and phenols, but not pesticides, were associated with lower blood pressure during pregnancy.	(181)
6 General metabolites of organophosphates. 7 Phenols. 10 Phthalate metabolites. 1 Nicotine metabolite.	154 Pregnant women. 152 Children, age 8.	Different LC–MS/MS methods were employed.	Assess variability of urinary concentrations of non-persistent chemicals by intraclass-correlation coefficients in participants from different Europeans countries.	A few dozen samples are required to accurately assess exposure over trimesters or months.	(182)
6 General metabolites of organophosphates.	116 Children, age 18–21 months	Extraction from diapers with acetone. SPE with Oasis WAX. HPLC-ESI-MS/MS.	Clarify organophosphate pesticide exposure in Japanese toddlers.	Statistical analysis confirmed the agreement between the DAPs concentrations in urine absorbed by diapers with urinary sampling.	(183)
6 General metabolites of organophosphates.	1,037 Children, age 16–23 months.	Extraction from diapers with acetone. SPE with Oasis WAX. HPLC-ESI-MS/MS.	Examine organophosphate exposure in toddlers and exposure-related behaviors.	Japanese toddlers were widely exposed to organophosphate pesticides.	(184)
4 General metabolites of organophosphates	222 Children from agricultural communities, age of 3-11 years.	SPE with C-18 Sep-Pak cartridges. UPLC-ESI-TQ.	Assess and correlate the exposure to organophosphate pesticides by DAPs in urine and hair from children from agricultural communities living near plastic-covered greenhouses in Almería (Spain).	The determinants of hair DAPs levels were identified. Whereas none of the predictors studied was significantly associated with urinary DAPs, except age.	(185)
Pyrethroids general metabolite 3-PBA.	80 Children, age 2–3 years.	Acid hydrolysis with HCl. LLE with n-hexane. Derivatization. GC–MS.	Identify associations for urinary concentrations of 3-PBA and cypermethrin hand wipe concentrations in children living in urban Bangkok (Thailand).	Gender presented a significant correlation with pesticide exposure, but walking in barefoot inside the household was the most determinant factor.	(186)
Pyrethroids general metabolite 3-PBA.	80 Children, age 2–3 years.	Acid hydrolysis with HCl. LLE with n-hexane. Derivatization. GC–MS.	Investigate the association between 3-PBA and GABA concentration in the urine of urban children from Bangkok (Thailand).	Use of insecticide coil allied to the habit of walking barefoot in households can increase exposure of children to pyrethroids. Resulting in alterations of the GABA levels.	(187)
Pyrethroids general metabolite 3-PBA.	21 Children with diagnosed with Autism Spectrum Disorder. 19 Children in the control group.	Acid hydrolysis with HCl. SPE with Strata X cartridge. Derivatization with HFIP and DIC. LLE with n-hexane. GC–MS.	Evaluate Pyrethroids exposure differences in children with autism spectrum disorder.	The levels of 3-PBA were only marginally higher (*p* = 0.054) in the group of ASD children.	(188)
7 Neonicotinoid pesticides.	46 Children, age 3-6 years	SPE with Presep RPP cartridges. HPLC-ESI-MS/MS.	Assess neonicotinoid exposure of children from Nagano (Japan) before, during, and after an area spraying.	Analyses showed that inhalation of neonicotinoid to control pine wilt disease in close areas were not associated to exposure, but the high detection frequency indicated contaminated food intake.	(189)
23 metabolites: -11 organophosphates -6 pyrethroids -6 herbicides from different classes	40 children	•DAPS Lyophilization. Derivatization to chloropropyl phosphate esters. GC–MS/MS. •Pyrethoid, specifics organophosphates. Enzymatic hydrolysis. SPE with Oasis HLB. HPLC-ESI-MS/MS and HPLC-APCI-MS/MS.	Evaluate the reduction of pesticide exposure in children due to consumption of organic diet.	Consumption of organic food was associated with reduced exposure to specific pesticides, 2,4-D and organophosphates.	(190)
9 Neonicotinoids and 4 metabolites	14 Children and adolescents, age 3–18 years	Protein precipitation with solvent. SPE. UPLC-ESI-MS/MS.	Determine if children’s cerebro-spinal fluid can be contaminated by neonicotinoids by analysing fluid, blood and urine from children at Switzerland.	All CSF and plasma samples were positive for at least one neonicotinoid. Moreover, 93% cerebro-spinal samples had N-desmethyl-acetamiprid.	(191)
Glyphosate	95 children, age 6–16 years	UPLC-MS/MS.	Determine glyphosate concentration in children from a rural community in Chapala Lakeshore (Mexico).	Urinary glyphosate level was significantly associated with season and age of children	(192)
Glyphosate and its metabolite AMPA	149 Children, age 7–10 years. 97 Adolescents, age 12–15 years.	Evaporation. Derivatization with 2,2,2–trifluoroethanol and trifluoroacetic anhydride. GC–MS/MS.	Estimate glyphosate and AMPA exposure in children and adolescents living in agricultural areas at Prekmurje (Slovenia).	Glyphosate and AMPA were detectable in 27 and 50% of the urine samples, respectively, in the first sampling. No participant exceeded the proposed reference value of 0.8 ng·mL^−1^ for glyphosate.	(193)
16 Pesticides biomarkers	281 Children and adolescents from agricultural communities, age 5–15 years.	HPLC-ESI-TQ.	A cross-sectional study with children under 15 years of age in agricultural communities of Ahuacapán (Mexico).	At least 2 pesticides of 16 were detected in all participants.	(194)
14 Pesticides biomarkers	1,060 Adolescents, age 17–21.	Different LC–MS/MS methods were employed.	Evaluate temporal trends and pesticides exposure in adolescents from Scania (Sweden)	Adolescents are frequently exposed to low concentration of pesticides.	(195)
Glyphosate	71 Mother–child pairs.	HPLC-MS/MS.	Evaluate the association between urine GLY level with pregnancy length and fetal growth in pregnant women from Indiana.	Higher GLY urinary levels were significantly correlated with shortened gestational lengths	(196)
Atrazine and 7 metabolites	174 Mother–child pairs in the case group. 195 Mother–child pairs in the control group.	SPE with Strata X-C cartridge. HPLC-APCI-MS/MS.	Assess the association between atrazine exposure during pregnancy and age of menarche in the offspring.	The association was significant for the most detected metabolite diaminochlorotriazine for the subset of girls with complete confounder information	(197)
4 general organophosphates metabolites: DMP, DEP, DETP, and DEDTP.	50 Mother–child pairs.	Acid hydrolysis with HCl. SPE with Bond Elut PPL. Derivatization with 1-chloro-3-iodopropane.	Examine Thai maternal exposure to organophosphate pesticides during pregnancy and offspring behavioral development.	Prenatal exposure was associated with negative effects over infant cognitive and motor development.	(198)
TCP, specific metabolite of the organophosphate chlorpyrifos.	377 Mother–child pairs.	Acid hydrolysis with HCl. LLE with hexane and MTBE. SPE with basic silica cartridge. Derivatization with BSTFA. LVI-GC–MS–MS analysis.	Evaluate associations between prenatal and postnatal exposure to chlorpyrifos with the neurodevelopment of children from agricultural communities at Jiangsu Province (China).	Exposure to chlorpyrifos during early childhood was associated with neurodevelopmental effects.	(199)
6 General organophosphates metabolites	310 mother–child pairs	GC–MS/MS	Assess prenatal and postnatal organophosphates exposure with birth outcomes and infant neurodevelopment.	Prenatal and postnatal DAPs were significantly associated with negative effects in the infant neurodevelopment.	(200)
6 General organophosphates metabolites	231 mother–child pairs	SPE. LC–MS/MS.	Identify detrimental effects over the cognitive function of children due to pregnancy exposure to organophosphate pesticides.	Although significant associations were identified, no clear association was obtained between prenatal exposure to DAPs and detrimental cognitive effects in the offspring.	(201)
6 General organophosphates metabolites	601 mother–child pairs	GC–MS/MS	Investigate the association between prenatal exposure to organophosphate pesticides and traits of autism spectrum disorders.	Prenatal concentrations of DAPs were associated with poorer reported social behavior.	(202)
6 General organophosphates metabolites	708 mother–child pairs	GC–MS/MS	Assess prenatal DAP concentration and Intelligence Quotient at nonverbal children of 6-year-old.	Consistent associations were not obtained.	(203)
6 General organophosphates metabolites.	784 Mother–child pairs.	Enzymatic hydrolysis. Derivatization with PFB-Br. LLE with dichloromethane and hexane. GC–MS/MS.	Examine whether mother exposure to organophosphate pesticides is related to ADHD and autistic traits in young children from Rotterdam (Netherlands).	There were no associations between maternal urinary concentrations of organophosphates with ADHD and autistic traits in children.	(204)
6 General organophosphates metabolites	784 mother child-pairs	GC–MS/MS	Assess exposure to organophosphate pesticides and fetus *in utero* and at delivery size.	Maternal prenatal DAPs concentrations were associated with decreased fetus length and weight *in utero*, but not at delivery.	(205)
6 General organophosphates metabolites	1,143 mother–child pairs from 3 cohorts	GC–MS/MS	Identify associations between DAPs concentrations and impacts over fetal growth.	DAPs concentrations were associated with decreased birth length for non-Hispanic black women	(206)
Carbofuranphenol, a metabolite of the carbamate carbofuran	1,100 mother–child pairs.	Acid hydrolysis with HCl. LLE with hexane and MTBE. SPE with basic silica cartridge. Derivatization with BSTFA. LVI-GC–MS–MS analysis.	Assess association between prenatal carbofuranphenol exposure and birth outcomes in pregnant women from Sheyang County (China).	Prenatal exposure to carbofuranphenol might have negative effects on fetal development.	(207)

### 4.1 General population exposure

Biomonitoring of the general population serves various purposes, such as assessing pharmacokinetics through residual pesticide determination in urine. For instance, a study evaluated dermal absorption from clothing treated with permethrin, commonly used as an insect repellent, revealing varying elimination rates among the six participants and establishing a half-life of 56 h (129).

Comparative analysis between urine and hair was another focal point in biomonitoring, especially concerning endocrine disruptors. A study employed repeated urine sampling to assess 16 phthalates, 4 bisphenols, and 8 pesticides. Findings suggested that while biomarkers were more frequently detected in urine, hair analysis yielded more consistent results (130).

The issue of public exposure to contaminants is increasingly pressing modern society, requiring a comprehensive global overview. In a small-scale study conducted in Kinshasa, Democratic Republic of Congo, involving 15 participants and pesticide determination. Urine analysis revealed detection frequencies exceeding 93% for glyphosate, 3-PBA, and TCP, with median concentrations of 0.2, 2.3, and 4.4 ng·mL^−1^, respectively (131).

Likewise, a pilot study conducted in Ireland delved into glyphosate exposure, unveiling a median concentration of 0.9 ng·mL^−1^ with a detection frequency of 20%, despite using a relatively elevated detection limit of 0.5 ng·mL^−1^ (133). In a separate exploratory investigation on glyphosate exposure in Portugal, encompassing 79 participants, a 73% detection frequency was noted, accompanied by a median concentration of 0.09 ng·mL^−1^. Furthermore, AMPA was detected in 97% of participants, with a median concentration of 0.10 ng·mL^−1^ (134).

Widespread exposure to low concentrations was observed in a study investigating neonicotinoid pesticides among 75 participants from Kumasi, Ghana. Approximately 92% of the participants were found to be exposed to multiple active ingredients (135).

Another study, conducted in North Carolina, United States, evaluated the exposure to pyrethroids in 50 residents over a six-week monitoring period. Analysis of urinary 3-PBA at weeks 1, 2, and 6 revealed a poor Intraclass Correlation Coefficient, indicating that a single measure of urinary 3-PBA was insufficient to characterize an individual’s average exposure effectively (136). Subsequently, examination using 24-h sampling demonstrated a positive association between exposure and factors such as outdoor activity, creatinine levels, and consumption of coffee and bread (137).

In a comparative study in North Carolina and New Jersey, United States, 30 individuals and their canine companions were enlisted to examine pesticide levels in both urine samples and silicone wristbands. The findings revealed that 7 out of 15 pesticide residues were detected in over 50% of the participants and their dogs, implying a parallel exposure to pesticides between humans and their animals (140).

A more comprehensive study involving 306 young men in Poland assessed four pyrethroid metabolites. Detection frequencies of 76% for permethric acid and 69% for 3-PBA were observed, with respective medians of 0.27 and 0.23 ng·mL^−1^. Higher exposure was associated with pyrethroid use in pet dogs and the consumption of seeds, nuts, and juice (138). In another investigation, 127 adult caregivers of children were assessed, revealing a detection frequency of 89% for various pesticides, with medians ranging from 0.3 to 3.4 ng·mL^−1^. Notably, exposure determinants varied for each active ingredient (139).

The exposure of Australian residents to organophosphates, pyrethroids, and phenoxyacetic acids was evaluated in a study involving 100 participants. Median concentrations for pesticides ranged from <0.1 to 36.8 ng·mL^−1^. Interestingly, for five organophosphate metabolites, higher exposure was observed in the youngest and oldest age strata (141).

Assessing urinary pesticide concentrations poses inherent challenges, particularly in studies involving large cohorts. However, this type of epidemiological study is widely recognized as the most appropriate method for obtaining reliable results and deriving meaningful conclusions. Consequently, methodologies for estimating exposure are pivotal in these investigations. In line with this, the Multi-Ethnic Study of Atherosclerosis conducted an assessment of organophosphate metabolite concentrations among its 4,466 participants. This endeavor produced consistent values, further validated within a subcohort comprising experimental data using GC–MS/MS for 240 individuals (142).

The effects of organophosphate pesticides on lung capacity were investigated in the Canadian Health Study cycle, a significant large cohort involving 4,446 participants. Residual urinary concentrations of DAPs were associated with reduced lung capacity, highlighting the adverse health effects associated with exposure to these pesticides (143). Subsequently, a global assessment was conducted with 322 participants from 8 different countries, targeting 10 analytes including 4 organophosphate metabolites, 2 phenoxyacetic acids, and 4 pyrethroid metabolites. Pesticide traces were detected in all urine samples with median ranging from 7.1 to 28.9 ng·mL^−1^. Regardless of regional exposure levels, pesticides represent a significant health issue that need prompt and effective intervention (144).

These large-scale studies afford the statistical power required to discern associations between pesticide exposure and health outcomes. In this vein, a study encompassing 2,116 participants from the general population of the United States was conducted to investigate pyrethroid exposure. Participants were stratified into tertiles based on 3-PBA medians of 0.1, 0.3, and 1 ng·mL^−1^. Exposure was correlated with overall mortality and cardiovascular disease mortality, although no discernible association with cancer was identified (145). Given the intricate nature of cancer as a broad and heterogeneous group of diseases, a detailed approach to its investigation is probably imperative.

The exposure of the United States population to neonicotinoid pesticides was examined in a cohort of 3,038 participants. Approximately half of the population had recently been exposed to at least one neonicotinoid pesticide. The 95th percentiles for N-desmethyl-acetamiprid and 5-hydroxy imidacloprid were determined to be 1.3 and 1.4 ng·mL^−1^, respectively, with young children and individuals of Asian descent exhibiting higher levels of exposure, although causal relationships remain unclear (146).

Socioeconomic characteristics have been extensively studied in relation to pesticide exposure associations. However, the impact of genetic variations, such as the polymorphism of the FLG gene encoding the filaggrin protein in the skin barrier, cannot be overlooked. For instance, null carriers of this gene were found to be more susceptible to skin exposure to pyrimethanil and two other environmental contaminants in dermal exposure experiments (147).

Research on the general population has predominantly centered on biomonitoring residual pesticide concentrations and identifying exposure determinants. These investigations aim to identify factors contributing to heightened exposure, encompassing objectives such as assessing dermal absorption, determining optimal sampling periods, utilizing urine and hair samples, and investigating the genetic dimensions interconnected with socioeconomic characteristics.

Notably, national and regional biomonitoring programs are the primary tools for assessing the general population and supporting chemical policies (208). These initiatives emphasize evaluating previously unidentified adverse effects of currently used pesticides and monitoring restricted or banned active ingredients (209). Additionally, they contribute to establishing human biomonitoring guidance values, which are crucial for identifying population groups at risk of negative health outcomes (210).

### 4.2 Occupational exposure

Occupational exposure to pesticides has been linked to a variety of adverse health effects, spanning noncommunicable diseases such as cancer, Parkinson’s disease, Alzheimer’s disease, as well as conditions like obesity and diabetes. In this context, extensive studies across various labor-related sectors are imperative to fully understand the scope and impact of these associations (211, 212).

#### 4.2.1 Farmers and agricultural communities

Population groups have undergone scrutiny concerning pesticide exposure in connection with agricultural practices. For example, occupational exposure among 58 residents of an agricultural community engaged in tulip cultivation in Netherlands was examined for five pesticides. The detection frequencies of chlorpropham and tebuconazole were 94 and 86%, respectively, with median concentrations of 0.8 μg·g creatinine^−1^ and 0.5 μg·g creatinine^−1^. Moreover, heightened exposure was observed during the application season among both farmers and non-farming families, suggesting that proximity to spraying areas should be considered a risk factor (149).

Furthermore, exposure to miconazole, a triazole fungicide, was assessed by analyzing monohydroxyl-miconazole (PEN-OH) and carboxyl-miconazole levels in urine samples collected from 22 pesticide applicators before, during, and after spraying activities. PEN-OH was detected in all samples, with median concentrations ranging between 8.0 and 27.6 ng·mL^−1^, and significant occupational exposure associated with both current body burden and total exposure levels (150).

Mancozeb, another fungicide widely used in agriculture, was the focus of an occupational exposure assessment among tractor operators in Italian vineyards. Pre-exposure median concentration of ETU was measured at 0.6 ng·mL^−1^, escalating to 1.9 ng·mL^−1^ post-application. Exposure determinants included the type of tractor cabin (open or closed) and the usage of Personal Protective Devices such as coveralls and gloves (151).

Subsequently, a study aimed to compare estimation strategies for actual exposure by determining ETU levels in urine, clothes, skin pads, and hand wash. Findings indicated that estimations based on the actual exposure period in hours and the Fixed Fractional Approach correlated more closely with urine levels than hand washing. Notably, over 90% of the exposure was attributed to hand contact (152). Furthermore, the establishment of the Biological Exposure Limit of 10 μg ETU·kg of body weight^−1^ in the 24-h post-exposure urine was based on skin exposure data collected from field experiments, using patches and urine sampling from 16 participants (153).

A study addressing occupational exposure resulting from neonicotinoid spraying was also presented. Imidacloprid was detected in all 43 participants, with a pre-application median concentration of 2.79 μg·g creatinine^−1^, raising to 10.52 μg·g creatinine^−1^ post-application (154).

Some studies examined crucial historical moments, such as the assessment of glyphosate exposure among rural populations in Wisconsin, United States, in 1997 and 1998, following the introduction of glyphosate-resistant soybeans in 1996. Glyphosate and AMPA concentrations were determined, with medians of 4.0 ng·mL^−1^ for glyphosate and 4.1 ng·L^−1^ for AMPA. Remarkably, non-applicators showed no detectable glyphosate concentration, while applicators exhibited a detection frequency of 39%, underscoring the occupational nature of the exposure (155).

Furthermore, toxicokinetics of glyphosate were investigated in 59 Thai farmers before spraying, and up to 72 h post-application. Among one-time sprayers, the average urinary glyphosate concentration measured 27.4 ng·mL^−1^, markedly higher than values reported in other countries (156). Subsequently, the impact of glyphosate application on oxidative stress, inflammation, and lung function was scrutinized in other 180 Thai farmers. Serum analyses revealed elevated levels of malondialdehyde, indicative of oxidative stress, alongside reduced glutathione levels and compromised lung function parameters (157).

Amidst ongoing debates surrounding the health effects attributed to glyphosate, recent studies have shed light on the toxic impacts of its co-formulants polyoxyethyleneamine (POEA), leading to fatalities among rats in experimental setups, prompting significant concern (213, 214). Further investigations have revealed that both POEA and the isopropylamine (IPA) salt of glyphosate infusion can disrupt hemodynamics and result in fatalities in piglets, in contrast to glyphosate in NaOH base, which did not exhibit similar effects (215).

A diverse range of alternative surfactants different from the traditional POEA have emerged in glyphosate formulations. Within this context, a comparative cytotoxicity study conducted with human cell lines revealed that alkyl polyglucosides exhibited the least toxicity. Following closely were polyethoxylated alkyl phosphate ethers and quaternary ammonium surfactants, while polyethoxylated tallow amines showed relatively higher toxicity levels (216).

In addressing the specific instance of acute ocular toxicity reported among glyphosate applicators in the past, formulations have been introduced that combine POEA surfactants with compounds aimed at reducing eye irritation. However, opting for less aggressive surfactants, such as propoxylated quaternary ammonium surfactants, is considered the optimal choice (217). While co-formulants have traditionally been viewed as inert substances, multiple scientific studies have highlighted the combined toxicity of adjuvants, underscoring the imperative for further research into these supposedly inactive components (218).

Occupational exposure to organophosphates has garnered significant attention in recent studies. In one investigation conducted in agricultural communities in South Tyrol (Italy), 28 farmers and 43 non-farmers were assessed for exposure to chlorpyrifos. During the application season, both farmers and residents exhibited elevated levels of TCP, with levels of 6.80 μg·g creatinine^−1^ for farmers and 6.73 μg·g creatinine^−1^ for non-farmers, compared to the non-application season where levels dropped to 2.54 μg·g creatinine^−1^ and 3.22 μg·g creatinine^−1^, respectively. This observation suggests that indirect contact to spraying significantly contributes to the overall exposure (158).

In another study focusing on organophosphate pesticide sprayers, 80 applicators and a control group of 90 non-occupationally exposed participants from Greece were evaluated for 4 general metabolites. The median concentration of DETP was found to be 4.06 μg·g creatinine^−1^ in participants with allergic rhinitis compared to 1.97 μg·g creatinine^−1^ in those without, indicating a potential association between these compounds and increased case numbers (160).

Furthermore, an analysis of urinary concentrations of 6 specific organophosphate metabolites and two general pyrethroid metabolites was conducted on 48 farmers and 77 urban individuals from Spain. The findings revealed that farmers exhibited approximately twice the exposure to these pesticides compared to non-occupationally exposed populations, with concentrations for 2-(diethylamino)-6-methyl-4-pyrimidinol at 1.7 ng·mL^−1^ and 0.81 ng·mL^−1^, 4-nitrophenol at 2.3 ng·mL^−1^ and 1.3 ng·mL^−1^, TCP at 4.2 ng·mL^−1^ and 2.2 ng·mL^−1^, and 3-PBA at 2.4 ng·mL^−1^ and 1.1 ng·mL^−1^, respectively (161).

In a more complete approach combining urinary pesticide residue determination and epigenomic investigation, indigenous Huichol farmers in Mexico occupationally exposed to organophosphate pesticides were assessed by methylation profiling of CDKN2A and CDKN2B genes, alongside the determination of six general metabolites of organophosphates in urine samples. The study encompassed 160 farmers and 30 non-farmers with similar levels of DAPs in both, indicating that the primary source of contamination stemmed from their living environment or ingested food (164).

In a study conducted in Thailand, involving 79 pesticide sprayers, the pre and post-application seasons were evaluated for 6 organophosphate metabolites. Significantly elevated residue levels were noted during the spraying season, coinciding with markedly lower cognitive scores among the participants (162).

The determination of a variety of active ingredients was employed to tackle the epidemic of Mesoamerican nephropathy in northwest Nicaragua. This cohort encompassed 350 young adults from rural communities, identifying 3-PBA, 2,4-D, DCCA, ETU, hydroxy-tebuconazole, and 3,5,6-trichloro-2-pyridinyl at detection frequencies exceeding 90%, but data did not reveal any correlation between these exposure biomarkers and the incidence of this disease, showing that this exposure might be not related with this health condition (148).

In various studies, pesticide has been linked to the development of chronic diseases. Occupational exposure, characterized by prolonged contact with pesticides, has been notably associated with an elevated incidence of dementia, particularly Alzheimer’s disease (219). This correlation is underscored by the connection between pesticide-active ingredients and phenomena such as oxidative stress, neuronal impairment, and cognitive dysfunction (220). Notably, paraquat, a common active ingredient, has been prominently linked to an increased risk of Parkinson’s disease. At the same time, even rotenone, a plant-derived pesticide used by home gardeners, has been implicated in this neurological disorder (221).

It is crucial to recognize that oxidative stress, induced by many pesticides, is a common denominator in various health issues, including cancer and neurodegenerative diseases (222). Although the precise mechanisms connecting pesticides to cancer remain unclear, being likely influenced by complex factors, numerous epidemiological studies have observed associations between pesticide exposure and various human cancers (223).

Moreover, pesticide levels have been associated with congenital disabilities, reproductive abnormalities, and fatalities (224). However, establishing a definitive link between chronic diseases and pesticide exposure, like other environmental risk factors, often yields inconclusive findings. This inconsistency may be attributed to challenges in obtaining precise data regarding individuals’ exposure levels, which are frequently inferred rather than directly measured (225). However, employing a combination of both estimation and direct measurement in modeling would likely provide a more robust approach.

It is important to note that the presented literature highlights that farmers and agricultural communities face heightened exposure to pesticide concentrations, warranting special attention due to potential health ramifications stemming from this increased contamination.

#### 4.2.2 Other occupations

Although much of the research documented in the literature has focused on agricultural communities, it’s important to recognize that other occupations face significant exposure to pesticides, including workers in flower-related industries and pest control.

The cultivation of flowers presents a high potential for pesticide contamination. For instance, a study conducted in Mexico assessed organophosphate pesticide exposure in 117 flower growers. The investigation found that over 90% of participants showed detectable levels. Notably, individuals working in greenhouses exhibited higher levels of exposure compared to those working outdoors, emphasizing the importance of personal protective equipment (165).

Similarly, occupational exposure among florists handling collected flowers was investigated in Belgium. Analysis of 56 workers revealed an average of 8 pesticide residues per urine sample, with an average total concentration of 4.3 mg.g creatinine^−1^. In contrast, the non-occupationally exposed group showed significantly lower levels at 2.0 mg.g creatinine^−1^. Therefore, florists exhibited higher concentrations and a greater variety of active ingredients in their exposure profiles (166).

Pest control workers were studied for their exposure to organophosphate insecticides. A study involving 230 Japanese employees explored gene polymorphism in relation to urinary concentrations of organophosphate metabolites. While no significant associations were found between metabolite concentrations and single nucleotide polymorphisms, there were noteworthy inverse associations observed between DAPs concentrations and the activities of fenitrothion oxidase and arylesterase (167).

Occupational exposure among amenity horticulturists was thoroughly examined, specifically targeting glyphosate. A cohort of 20 workers underwent monitoring via multiple spot urine samples. The resulting median glyphosate concentration was recorded at 1.9 ng·mL^−1^, with the peak excreted concentration observed after 3 h of exposure (168). Furthermore, associations were established between glyphosate and fluroxypyr urinary levels and various work-related factors, including breaks, extended tasks, and the utilization of controlled droplet applicators or boom sprayers (132).

Pyrimethanil and its metabolite 4-hydroxypyrimethanil were targeted for biomonitoring in both the general Swedish population and occupationally exposed horticulturists. Among 413 individuals, approximately 50% exhibited contamination, with a median hydroxypyrimethanil concentration of 0.1 ng·mL^−1^. In contrast, among 18 assessed horticulturists, a much higher detection frequency of 96% was observed, with a median concentration of 8 ng·mL^−1^ (169).

These findings suggest that various occupations can lead to increased contamination levels, as evidenced by the general population typically displaying lower concentrations of biomarkers for different active ingredients. In light of this, biomonitoring equivalents play a crucial role. These data are defined as the concentrations of chemicals in biological samples consistent with existing guidance values, such as reference doses, reference concentrations, minimal risk levels, or tolerable daily intakes, indicating the necessity for further assessment of exposure sources (210, 226, 227).

### 4.3 Reproductive disorders and early stages of human life

Pesticide exposure was also assessed with a focus on reproductive disorders and early stages of life, targeting various adverse health effects.

#### 4.3.1 Reproductive disorders

The impact of pesticides on fertility prior to conception was investigated, particularly focusing on organochlorine pollutants and organophosphate pesticides in men and women. For example, the levels of op’-DDD (median: 0.018 ng·mL^−1^) and pp’-DDE (median: 0.044 ng·mL^−1^) in urine of 111 infertile men from Pakistan showed a negative correlation with sperm motility in men. While serum is typically used to assess organochlorine pesticide exposure, this alternative sampling can offer significant results (170).

In another investigation, involving 159 men from United States, exposure to organophosphate pesticides was assessed through the detection of 6 general metabolites, with DAPs being detected in all cases, averaging at 188 nmol·mL^−1^. Positive associations were found between DMTP, DMDTP, DEP, and DETP with sex chromosome disomy, whereas an inverse association was observed for DMP, suggesting the occurrence of complex relationships (171).

Both men’s and women’s infertility are equally significant and warrant attention. Thus, a cohort comprising 594 reproductive-age women from USA was analyzed to investigate the connections between endometriosis and 11 pesticides, known as endocrine disruptors. The odds ratios for endometriosis diagnosis notably escalated between the first and fourth quartiles of exposure to diazinon (OR = 1.89) and chlorpyrifos (OR = 1.65), underscoring a correlation between these pesticides and endometriosis (172).

Furthermore, exposure to organophosphate and pyrethroid pesticides prior to conception was evaluated in a cohort of 614 women in China. This study found that higher levels of DETP and 3-PBA were associated with prolonged time to pregnancy and increased risk of infertility (173). In the same context, an investigation also conducted in China examined the impact of organophosphates on *in vitro* fertilization outcomes. Women in the highest exposure quartile exhibited reduced odds of successful implantation, clinical pregnancy, and live birth (174).

These investigations highlight the significance of assessing pesticide exposure to reproductive health, revealing noteworthy adverse effects that require attention. These studies have shed light on various reproductive disorders discussed in the literature, implicating different active ingredients as endocrine-disrupting chemicals capable of prolonging the time to conceive, and increasing the risk of infertility and congenital disabilities in women (12). Furthermore, research suggests that pesticide exposure in males can adversely affect sperm and semen quality, leading to various reproductive disorders (14).

#### 4.3.2 Pregnancy

Pregnancy represents a critical period of vulnerability to pesticide exposure, prompting focused investigation into its impact on gestation. Within this perspective, a study examined sampling periods to assess 6 general metabolites of organophosphates in a cohort of 62 pregnant women. Median concentrations ranging from 0.05 to 3.6 ng·L^−1^ were observed, with afternoon sampling showing stronger correlation with multiple sequential samples than first-morning void, suggesting its preferential consideration (175).

The variability of excretion for non-persistent chemicals and optimal urine sampling strategies were also assessed in a cohort comprising 154 pregnant women and 152 children (8 years old) from various European countries. Phthalates, phenols, DMTP, and DEP were detected in over 87% of samples, and the sampling scheme indicated that three daily pools of two urine samples each were sufficient for weekly exposure determination, resulting in reduced variability for biomonitoring (182).

Another pertinent area of inquiry was identifying exposure determinants, particularly during pregnancy among occupationally exposed farmers. This investigation involved 50 participants in Thailand and conducted a serum metabolome-wide association study on chlorpyrifos exposure. The median urinary concentration of TCP was found to be 2.39 ng·ml^−1^. Additionally, exposure correlated with indicators of oxidative stress, cellular damage and repair, as well as systemic inflammation, potentially posing adverse health effects on the fetus (176).

In a comprehensive investigation, a study evaluated the exposure of 573 Spanish pregnant women to 4 general and 4 specific metabolites of organophosphate pesticides. The detection frequency of these metabolites ranged from 6 to 59%, with a median concentration sum of 3 ng·mL^−1^. Subsequently, exposure was associated with factors such as fruit and vegetable intake, pre-pregnancy body mass index, and smoking habits during pregnancy (177).

Furthermore, the assessment of pyrethroid exposure was conducted on 480 pregnant women from non-rural areas of Yunnan, China. Detection frequencies for 4F-PBA, 3-PBA, and DBCA were 96, 90, and 72%, respectively, with a total pyrethroid median of 1.38 ng·mL^−1^. In addition, exposure positively correlated with the consumption of bananas, oranges, and the variety of fruits regularly consumed (178).

Exposure to both organophosphates and pyrethroids insecticides was investigated in a cohort of 30 pregnant Taiwanese women. The detection frequency of DMTP was 33%, with a median concentration of 1.19 ng·mL^−1^, while DCCA, a pyrethroid metabolite, showed a detection frequency of 73% with a median concentration of 11.31 ng·mL^−1^. Correlations between parent compounds in blood and their metabolites in urine indicated regular exposure, corroborated by the known use of these active ingredients in domestic insecticide products (179).

Further studies encompassing a wider array of contaminants were conducted. For example, 200 pregnant women from Denmark were assessed for exposure to parabens, phthalates, and phenols, including two metabolites of phenoxyacetic herbicides. Detectable levels were observed in nearly all participants, suggesting multiple combined exposures. Therefore, current risk assessments often overlook simultaneous exposure to several contaminants, potentially underestimating the health effects on fetuses (180).

Within the sphere of health effects, hypertensive disorders are among the leading causes of maternal and neonatal mortality and morbidity. Thus, a study involving 152 pregnant women from three European regions investigated blood pressure alterations and exposure to non-persistent chemicals. Although detection frequencies for organophosphate metabolites ranged from 66 to 98%, with medians of 1.4–3.9 μg·g creatinine^−1^, disorders were not associated with these exposures, showing that some hypothesis may be rejected by data (181).

The vulnerability of fetuses to environmental contaminants, including pesticides, has sparked concerns regarding exposure to various chemicals, making it an exceptionally pertinent research area that demands careful attention. Moreover, pregnancy represents a unique period characterized by biological changes that can heighten susceptibility to environmental chemicals and increase health risks for women (228).

Within this framework, pesticides have been associated to the promotion of inflammation and oxidative stress, resulting in membrane damage, protein dysfunction, and DNA impairment. In addition, specific active ingredients have been identified as endocrine disruptors with the potential to impact fetal growth (229). Therefore, an increasing necessity exists for studies to thoroughly investigate the health impacts of pesticide exposure on pregnant women. Currently, these investigations have primarily concentrated on growth, immunological and neurobehavioral development, respiratory function, and hormonal balance (230).

#### 4.3.3 Childhood and youth

Children’s exposure to pesticides is a critical issue, especially during early childhood when even minimal exposure can have profound effects. Regarding this, a study encompassing 116 Japanese children investigated toddlers’ exposure to organophosphate pesticides through analysis of urine from diapers. Detection frequencies ranged from 49 to 100% for each DAP, with a median concentration of 137.6 nmol·L^−1^ (183). Subsequently, a larger study involving 1,037 toddlers reported a total median concentration of 120 nm·L^−1^. In conclusion, household products such as herbicides, insect repellent sprays, fragrances, and deodorants were identified as associated factors with DAPs levels (184).

The exploration of exposure determinants has been a focal point in certain studies focusing on children. In an investigation involving 222 children, both hair and urine analyses were employed to evaluate 4 DAPs. This study unveiled a detection frequency of 46% for at least one metabolite in urine and an impressive 99% in hair samples. Although distinct determinants were noted for hair analysis, none showed associations with urinary levels, except for age. Therefore, according to these data, the inclusion of hair analyses should be deemed essential in biomonitoring initiatives (185).

An assessment of 7 neonicotinoids focused on their use against pine wilt disease, which affects trees. Thus, 46 children in Nagano, Japan, were evaluated with 100% detection of neonicotinoid residues in urine. Air particulate and exposure association analysis suggested that the high detection frequency was primarily linked to dietary habits, rather than thiacloprid spraying in nearby areas (189). Regarding food intake, the impact of an organic diet was analyzed by assessing 23 compounds in 50 children from California, USA, over 16 consecutive days. While reduced concentrations of 2,4-D and organophosphate metabolites were observed due to ingestion of organic food, pesticide residues were still present, indicating other routes of exposure (190).

Glyphosate exposure was evaluated in 95 children and adolescents from agricultural communities in Chapala Lakeshore, Mexico. The detection frequency was 100% with a median of 0.37 ng·mL^−1^ for children from 6 to 16 years, and 0.03 ng·mL^−1^ for those between 3 and 6. In addition, season and age were significantly associated with determined contamination levels (192).

Furthermore, an investigation into the exposure determinants on agricultural communities involved the biomonitoring of glyphosate and its metabolite AMPA, in 149 children and 97 adolescents in Prekmurje, Slovenia. The detection frequencies were 27% for glyphosate and 50% for AMPA. The 95th percentile concentrations were ≤ 0.21 ng·mL^−1^ for glyphosate and ≤ 0.33 ng·mL^−1^ for AMPA. Increased exposure was correlated with the consumption of nuts and wholegrain rice, as well as elevated urinary levels of arsenic, lead, cobalt, zinc, and copper, indicating potential combined exposures (193).

The pyrethroid general metabolite 3-PBA was monitored in 80 children aged 2–3 years from Bangkok, Thailand, with a median concentration of 1.46 ng·mL^−1^ and a detection frequency of 92%. Exposure was linked to cypermethrin concentration in hand wipes, walking barefoot in the household, and gender (186). Subsequent research on the same cohort revealed a negative correlation between the median concentration of 3-PBA and gamma-aminobutyric acid, an inhibitory neurotransmitter, suggesting potential neurological consequences (187).

Also approaching health effects, a case–control study involving 40 children assessed the levels of exposure in participants with autism spectrum disorder (*n* = 21) compared to a control group (*n* = 19). The levels of 3-PBA were marginally higher in the case group, indicating a potential association, however a larger experimental design would be necessary for definitive conclusions (188).

Given that neonicotinoids bind to mammalian nicotinic acetylcholine receptors, researchers assessed the presence of 9 active ingredients and 4 metabolites in the cerebrospinal fluid (CSF) of 14 children and adolescents. Notably, 93% of CSF samples contained N-desmethyl-acetamiprid. Therefore, the broad exposure and detection of neonicotinoids even in CSF highlight the need for exposome studies to comprehend the risks of childhood cancers and other health consequences (191).

In a more comprehensive cross-sectional study encompassing 281 children under the age of 15 from two agricultural communities, Agua Caliente and Ahuacapán at Mexico, exposure to 16 pesticides was thoroughly investigated. Remarkably, every sample revealed detectable levels of at least two pesticides. Consequently, it is paramount to address potential long-term health effects stemming from concurrent exposure to diverse active ingredients (194).

An interesting temporal analysis was undertaken among Swedish adolescents, investigating 14 pesticides across the years 2000, 2004, 2009, 2013, and 2017. Despite participants displaying relatively low concentrations, with medians falling below 3.9 ng·mL^−1^, upward trends were discerned for 3-PBA, chlorpyrifos, pyrimethanil, and tebuconazole. Conversely, diminishing values were observed for chlormequat, thiabendazole, and ETU. These findings suggest a shift in pesticide usage habits and, consequently, exposure patterns (195).

Several studies have demonstrated that children and teenagers are exposed to various active ingredients. However, further research is needed to understand the associations with health outcomes and the impacts of observed contamination levels. Exposure biomarkers have significantly contributed to the study of pesticides and other environmental pollutants on childhood health, complementing traditional assessment methods such as questionnaires (231). Importantly, research suggests higher levels of exposure in children, possibly due to their smaller body mass, faster metabolism, increased ingestion of non-food items, and more outdoor activities on the ground, particularly in households where parents are occupationally exposed (232).

#### 4.3.4 Mother–child pairs

Mother–child cohorts have emerged as a crucial strategy in numerous studies aimed at investigating the health impacts on offspring, particularly in assessing exposure to environmental contaminants. For example, a study conducted in Central Indiana, United States, examined glyphosate exposure in 71 pregnant women, uncovering a detection frequency of 93% and a median concentration of 3.3 ng·mL^−1^. Notably, this exposure was significantly associated with shortened gestational lengths, underscoring its potential impact on maternal and fetal health (196).

In another example highlighting associations with health effects. *In-utero* exposure to atrazine was examined in a case–control study involving 369 mother–child (girls) pairs at 12 weeks. The metabolite diaminochlorotriazine (DACT) was detected in 58% of cases and was linked with early menarche, providing further insight into the potential impacts of environmental contaminants on reproductive health (197).

Assessment of organophosphate exposure during pregnancy was carried out in 50 mother–child pairs from Thailand. The median DAPs concentration at week 28 of gestation was 85.5 nmol·L^−1^, with a detection frequency of 96%. It is important to note that this exposure was correlated with reduced neuromotor scores at five months of age (198).

Evaluating a larger cohort, prenatal and postnatal exposure to the organophosphate chlorpyrifos was investigated in 377 mother–child (3-year-old) pairs from agricultural communities in Jiangsu province, China. Median concentrations of TCP in the urine of mothers and children were 5.4 ng·mL^−1^ and 5.3 ng·mL^−1^, respectively. In this study, postnatal exposure was negatively associated with children’s motor and social development, particularly in boys (199).

Similarly, an examination of 310 mother–child pairs with 2-year-old children, also from China, uncovered heightened risks of adverse neurodevelopmental effects. These findings emphasize the pressing need for public policy intervention to mitigate the deleterious impacts of DAPs on children (200). In contrast, in an evaluation involving 231 French mother–child pairs, determination of 6 DAPs at 19 weeks’ gestation and one sample from the child at 6 years old did not reveal clear detrimental effects due to organophosphate exposure, indicating the need for additional research (201).

Furthermore, the association between exposure to organophosphate pesticides and traits of autism spectrum disorder was examined in 601 mother–child pairs from the agricultural Salinas Valley in California, United States. Prenatal concentrations of DAPs were associated with poorer reported social behavior, indicating a potential link between organophosphate exposure and neurodevelopmental disorders (202).

Contrastingly, a study involving 708 mother–child pairs from Generation R in Rotterdam (Netherlands) did not consistently observe an association between prenatal DAPs concentrations and the intelligence quotient of nonverbal children at 6 years old (203). Also from Rotterdam, 784 mother–child pairs were evaluated based on maternal urinary concentrations of 6 general metabolites of organophosphate pesticides during gestation. Despite a median DAPs metabolite concentration ranging from 308 to 316 nmol·g creatinine^−1^, no significant association was found between attention deficit hyperactivity disorder and autistic traits during a 10-year follow-up (204).

In the Columbia and Mount Sinai birth cohorts in the United States, prenatal exposure to organophosphates was assessed through DAPs determination in relation to fetal growth. The findings revealed that DAPs concentrations were linked to decreased birth length among non-Hispanic black women (206). Moreover, in the Generation R study in Rotterdam, prenatal exposure to organophosphates was significantly associated with decreased fetus *in utero* weight and length, although not at delivery, suggesting intricate correlations (205).

A study conducted in Scheyang County, China, involving 1,100 pregnant women investigated the exposure to the carbamate carbofuran and its associations with birth outcomes. The median carbofuranphenol concentration obtained was 0.8 ng·mL^−1^, with a detection frequency of 100%. Exposure to carbofuran was found to be negatively associated with neonatal head circumference in boys, while positively correlated with the neonatal ponderal index (207).

Research has demonstrated that pesticides can permeate both the placental barrier and the fetal blood–brain barrier, thereby impacting the developing fetus when the mother is exposed (233). Given the potential long-term health implications in both childhood and adulthood stemming from early exposure to environmental contaminants during pregnancy, it is imperative to conduct longitudinal studies to obtain meaningful and significant results (234). These findings add to the mounting evidence indicating that exposure to environmental contaminants during early life can hinder proper development (235).

Mother–child pairs form a cohort category wherein attaining a substantial participant count for enhanced statistical power can prove challenging, although specific cohorts already encompass more than 1,000 participants (236). Yet, this research strategy is not represented well in low-and middle-income countries (237, 238). Nevertheless, epidemiological studies have globally highlighted adverse health effects in children resulting from pesticide exposure, although further studies employing consistent methodologies are still necessary to mitigate inconclusive results (239, 240).

The literature highlights the necessity of cohorts with sufficient participant numbers and gold standard analyses to establish correlations, conduct health risk assessments, and perform meaningful statistical interpretations. However, this demand poses a significant obstacle to research, particularly in low-income countries with scarce resources. Nonetheless, collaborative efforts must prevail to facilitate a comprehensive global assessment of pesticide effects.

## 5 Synthesis and future directions

Chromatography coupled with mass spectrometry has emerged as a powerful tool for biomonitoring pesticides and organic contaminants across various sample matrices. Among these matrices, urine, blood, serum, and hair, have been consistently preferred choices (52). Urine sampling has particularly garnered attention in both specific assessments and large-scale biomonitoring programs (146). While other matrices such as blood, plasma, serum, and hair have also been explored for similar purposes, urine data have been extensively utilized in biomonitoring efforts, informing the development of public health policies (241). This preference may be attributed to the easiness of sample collection and processing, the wealth of available information from diverse geographical regions, and participants’ potential apprehension towards blood withdrawal.

Sample preparation has been a focal point in several studies, with an emphasis on deconjugation methods (242). Acid hydrolysis with HCl and enzymatic hydrolysis using *Helix pomatia* glucuronidase have been commonly employed, since the determination of free metabolites has been addressed in only a few studies (243). While acid hydrolysis is cost-effective, it’s important to note that it may induce reactions in parent compounds and byproducts, potentially altering biomarker profiles (244). Consequently, enzymatic deconjugation has gained traction in recent years due to increased product availability and the use of milder conditions (245).

Various extraction strategies have been explored as alternatives for pesticides analysis. Classical LLE and SPE were extensively utilized in the early 2010s (43, 46, 69). Subsequently, miniaturized SPE employing milligrams of sorbent, liquid-phase microextraction, and SPME gained attention (103, 125). QuEChERS (116) and d-SPE (58) emerged as trends in sample preparation, offering reduced solvent usage and simplified sample handling. Additionally, other strategies such as DPX (59), MISPE (44), SFODME (45), DLLME-SFO (57), and VALLME (55) have been investigated, each presenting distinct advantages in terms of efficiency and environmental sustainability. In summary, these diverse options reflect the abundance of options available in contemporary sample preparation methodologies.

The initial methods predominantly concentrated on analyzing individual or a restricted set of active ingredients, frequently belonging to the same chemical class. This strategy was preferred owing to the shared physicochemical properties, which favored sample preparation and chromatographic separation (13, 88). Conversely, developing comprehensive multi-residue methods posed distinct challenges, especially with outdated systems known for their limited separation efficiency and detection capabilities, ultimately undermining analytical performance.

Another suitable choice was the type of inlet system, GC or LC. Gas chromatography is frequently considered a more robust and cost-effective technique, but the requirement of high temperatures reduces the possibilities regarding the analyte range (246). Furthermore, derivatization was another issue that have impacted extensive studies for most of the biomarkers from non-persistent pesticides at GC–MS and GC–MS/MS analyses, since this step of sample preparation requires time, additional human resources, and/or sophisticated automation. Therefore, avoiding these disadvantages, LC–MS/MS methods have been reported in increasing numbers, notably in recent years (123).

In the pursuit of miniaturization, most analytical methods from the 2010s employed HPLC as the separation technique. In later years, there was a transition to core-shell technologies, replacing porous particles to enhance efficiency and speed (107, 119, 247). Subsequently, the development of UPLC methods utilizing columns with particles smaller than 2.1 μm, coupled with improved mass spectrometers, became extensively explored for evaluating a wide array of analytes, particularly in strategies aimed at critical exposure assessment (125).

Allied with these advancements and faster switching speed, the scope of analysis has broadened to encompass pesticides from various chemical classes. This expansion now includes environmental contaminants arising from diverse sources, such as plasticizers, nicotine biomarkers, polycyclic aromatic hydrocarbons, and personal care products (68, 122, 182).

However, a noticeable distinction emerges between biomarkers characterized by significant hydrophilic properties, such as ETU, quaternary amines, DAPs, and aminophosphonic acids (50, 98), and hydrophobic analytes like phenols, pyrethroids, neonicotinoids, organophosphates, pyrazoles, and triazines (72, 117, 118). This differentiation arises from the specificities inherent in sample preparation and chromatographic separation techniques, owing to the divergent physicochemical properties. Consequently, this leads to the development of distinct multi-residue methods for each group of analytes.

Literature highlights the vital significance of carefully choosing exposure biomarkers as analytes and crafting methodologies with LOQs below 1.0 ng·mL^−1^. This necessity arises from the observation that numerous studies revealed concentration medians hovering around this threshold (139, 141). Therefore, ensuring heightened detectability is imperative for achieving statistically significant findings capable of driving subsequent correlations. Furthermore, leveraging state-of-the-art equipment typically augments the frequency of quantification, even when employing the same overall methodology, as updated systems offer superior instrumental sensitivity.

Additionally, different urine sampling schemes have been explored to enhance data reproducibility. While most studies utilize the assessment of the first urine void, the afternoon void has also emerged as a compelling option (175). Furthermore, more extensive sampling strategies, including 24-h sampling and repetitive collections over different days and weeks, have been investigated (168). However, such procedures necessitate additional efforts from data collection teams, particularly in extensive cohort studies. Despite this, studies on contaminants suggest that single-spot urine sampling may adequately reflect average population exposure for large cohorts (248).

Alternative sampling methods were explored, such as wristbands and hair analyses. In spite of that, urine sampling is the most employed matrix for non-persistent pesticide residues at large biomonitoring programs (71, 130, 140). In this context, a crucial aspect of urine assessment is normalization, as various methods have been proposed (249). Notably, creatinine has emerged as the most used normalization option in studies aiming to identify exposure determinants and associations with health effects (149, 204). It is important to note that some studies adopt both approaches, considering the interpretation of pesticide residue concentration in urine and creatinine-adjusted values. Furthermore, other normalization strategies can be assessed, like specific gravity and combined modeling (250).

While LC-TQ prevail as the predominant tool owing to its heightened detectability, there has been exploration into utilizing high resolution mass spectrometry (HRMS), such as LC-TOF and LC-Orbitrap, for residual pesticide determination in urine, showing relevant detection limits (97, 109, 115). Their application in post-targeted and untargeted analyses warrants special attention, as they facilitate the identification and quantitative estimation of unknown or unpredicted biomarkers, offering relevant additional data (110).

Human biomonitoring programs play a pivotal role in acquiring data that can inform policymakers in responding to environmental and human health threats, as evidenced by the banning several toxic active ingredients like organochlorine pesticides under the Stockholm Convention (251). Additionally, other active ingredients, such as organophosphate insecticides parathion and methamidophos, carbamates like aldicarb and carbofuran, and herbicides as paraquat (quaternary biphenyl ammonium) and 2,4,5-T (chlorophenoxyacetic herbicide) have been widely banned or highly restricted due to their high toxicity, which has been linked to various health problems (252).

Herbicides like atrazine (triadimefon) and glyphosate (aminophosphonic acid) are also under intense scrutiny. However, it’s imperative to acknowledge that pesticides should be evaluated within a comprehensive picture of the agricultural system, as banned active ingredients may be replaced by even more harmful compounds (253, 254).

The outlook of banned and currently used pesticides can vary from one country to another, posing a challenge for biomonitoring methods and strategies. Notably, obsolete pesticides have been detected as legacy chemicals or due to inappropriate application in various studies (255–257). This diversity in active ingredients, physicochemical properties, and formulations creates a complexity barrier for elucidating exposure levels, further complicated by differences in sampling strategies, analytical methods, and data presentation, necessitating standardization for direct comparison (258, 259).

Significant progress has been achieved in international and national legal frameworks concerning pesticide management. However, many existing laws may not fully align with the requirements of agreements or regional initiatives aimed at harmonizing regulations. Additionally, these laws often need to adequately integrate with updated legislation about environmental protection, chemicals management, and related areas (260).

Broadly, developed nations tend to impose more stringent regulations than developing countries, leading to disparities in pesticide legislation that pose technical barriers to commercial relations (261). As the globalization of food systems advances, the sharing of risks associated with safety, including exposure to pesticide residues, becomes more pronounced (262).

Counterfeit pesticides exacerbate the issue by flooding the market with substandard products. These inferior items are created through the adulteration of raw materials, inadequate purification processes, and low-quality solvents and packaging. Consequently, they introduce harmful impurities such as ethylmethanesulfonate, isomalathion, or nonylphenol ethoxylates (263).

Additionally, the smuggling of pesticides contributes to the widespread dissemination of banned and restricted active ingredients within agricultural practices (264, 265). These illicit activities pose significant challenges to biomonitoring programs and lead to substantial crop losses and environmental hazards. This black market, estimated to be worth around $5.4 billion, perpetuates risks to human health and ecosystems (263, 266).

Moreover, developing new analytical methods is crucial for evaluating non-conventional pest control products that could be incorporated into future human biomonitoring initiatives, despite these substances generally being deemed low in toxicity. These include a range of biopesticide products, such as microbial, semiochemicals, and pheromones, as well as plant extracts and oils of vegetal and mineral origins, and other natural solutions like diatomaceous earth (267).

Another category to consider is Plant-Incorporated Protectants, such as Cry proteins and double-stranded ribonucleic acid expressed in genetically modified crops (268). Additionally, there is a growing interest in nanopesticides, where active ingredients are applied with nanocarriers such as polymers, clays, and zein particles, presenting a promising new approach that may be addressed in future biomonitoring (269).

The determination of residual pesticides in urine serves various purposes, encompassing toxicological studies, assessment of pesticide exposure among the general population, and occupational exposure among farmers, pest control workers, horticulturists, and florists (166, 167, 270). In addition, it sheds light on the consequences of pesticide exposure during pregnancy and childhood, impacts on neurodevelopment, and the occurrence of reproductive disorders (199, 271).

Furthermore, recent research has underscored the importance of evaluating adjuvants, counterions, and co-formulants among exposed populations (272). Since several studies have highlighted the toxic effects, particularly those of POEA, necessitating stricter regulations and accurate formulation descriptions (217, 218).

Pharmacokinetic and occupational exposure studies have revealed that dermal absorption significantly contributes to contamination, alongside ingestion and inhalation, resulting in elevated exposure levels (88, 129, 149, 150, 153). Moreover, data concerning the general population indicate widespread detection of pesticide exposure to various active ingredients, emphasizing the critical issue of mixture effects. This complexity increase the challenge of establishing human biomonitoring guidance values, which remain imperative (131, 141, 172, 210).

Genotyping holds immense potential for future studies, as specific polymorphisms can profoundly influence human pesticide exposure. For example, variations in dermal polymorphisms of the FLG gene, responsible for encoding the filaggrin protein, can have significant implications (147, 167). Thus, epigenomic assessments, such as studies on gene methylation, also offer promise as a valuable tool in human biomonitoring, offering reduced variability in assessments, as in the correlation between the CpG site of the *CDKN2B* gene and work time in farmers (164). Furthermore, occupational pesticide exposure has been extensively linked to genomic instability and other genetic and epigenetics aspects (273).

In the context of occupational exposure, pesticide spraying and residing in agricultural communities are particularly pertinent, especially given concerns about pesticide overuse (274). While historical data suggested limited glyphosate exposure primarily among those involved in spraying activities, recent studies indicate that residing in rural areas can significantly elevate exposure levels (38). Consequently, individuals in rural settings may exhibit double the pesticide residue concentrations compared to urban populations, potentially leading to associated health effects (149, 158, 160, 161).

Flower cultivation and gardening activities have been identified as occupations with significantly heightened exposure risks. For instance, working within greenhouses has been shown to pose a greater risk compared to working in open fields for flower cultivation (165). Additionally, florists have been found to have elevated pesticide residue levels compared to the general population, suggesting that flowers can serve as a potential source of contamination (166). In this context, a noteworthy strategy was determining Equivalent Biological Exposure Limit, which is an important data for biomonitoring at work environment (154).

Different studies have reported adverse health effects stemming from inadequate use of personal protective equipment (275), also requiring the determination of biomonitoring equivalents to identify risky incidents and scenarios (210). Prolonged exposure to pesticides, as seen in certain occupational settings, has been linked to various chronic diseases, as different forms of cancer, cardiovascular issues, pulmonary diseases, as well as Parkinson’s and Alzheimer’s disease, spark significant concerns regarding the health impacts on workers, particularly in advanced age (251, 276, 277). Thus, there is a pressing need for comprehensive investigations utilizing both direct and indirect methods of exposure level determination, as discussed herein.

The impact of pesticide exposure on fertility in both women and men deserves comprehensive evaluation, uncovering adverse effects. For instance, there is emerging evidence suggesting a potential correlation between exposure to diazinon and chlorpyrifos to the development of endometriosis (172). In men, heightened levels of pesticide residues were noted in individuals experiencing infertility, notably with HCB showing an association with reduced sperm motility (170). Furthermore, specific organophosphate metabolites have been positively linked to disomic rates, highlighting the significance of further research in this area (171).

The escalating global concern over the impact of pollution on children’s health and development prompted significant attention. In response, the Miami Declaration was formulated during the 1997 G8 Meeting, involving key nations such as the United States, Canada, France, the United Kingdom, Germany, Italy, Japan, and Russia. This landmark declaration was crafted with the intention of advancing research on children’s environmental health, emphasizing the necessity for biomonitoring studies and programs (278).

The susceptibility of fetuses to environmental contamination has been extensively assessed through the inclusion of pregnant women in research, with follow-up extending from birth into adolescence. Various studies have aimed to pinpoint exposure determinants to mitigate individual contamination. However, most findings have underscored associations with factors such as fruit and vegetable consumption, and body mass index. Thus, addressing these concerns ideally involves opting for organic foods and maintaining a balanced diet (177, 178).

Additionally, toddlers’ exposure has been evaluated through urine analysis from diapers. Regarding this, widespread exposure to organophosphates and other pesticides has been noted, alongside exposure determinants such as household products like pesticides, fragrances, and deodorants (183, 184). Regarding health effects, research has revealed associations between atrazine exposure during pregnancy and early menarche in offspring (196, 197). Similarly, glyphosate exposure has been significantly linked to shorter gestational periods, while prenatal exposure to carbofuran has shown adverse health effects in baby boys (207), emphasizing the relevance of such investigations.

Nevertheless, it’s crucial to recognize that certain hypotheses concerning adverse health effects have not been validated. For instance, exposure to organophosphate pesticides showed no correlation with lower blood pressure during pregnancy (181) or with ADHD and autistic traits in children (204). Conversely, prenatal exposure has been linked to adverse effects on infant cognitive and motor development (198, 199). Hence, it is imperative to implement diverse strategies and validation studies to confirm associations and draw comprehensive conclusions.

Recent advancements in chromatography-mass spectrometry for urine analyses have facilitated the development of multi-residue methods capable of simultaneously determining several pesticide residues from various chemical and use classes. These innovative approaches can be employed in studies investigating the health risks associated with exposure to mixtures, including pesticides and other relevant environmental contaminants, thereby providing a more holistic understanding of human biomonitoring.

The variability in urine sampling methods, coupled with the need for rigorous numerical treatment, highlights the critical importance of recruiting a substantial number of participants to ensure the attainment of statistically significant results. Moreover, it is imperative to validate these findings across diverse demographic groups to guarantee their robustness and applicability.

In this regard, integrated strategies that foster collaboration among international research groups and biomonitoring programs play an essential role. By leveraging such efforts, we can maximize the utilization of available resources and significantly enhance the efficiency of initiatives for health risk assessment.

## Author contributions

WB: Writing – original draft, Writing – review & editing. FL: Writing – review & editing. AS: Writing – review & editing. HS: Writing – review & editing.
